# Supplementary material for: Emerging dominance of summer rainfall driving High Arctic terrestrial-aquatic connectivity
Source: Nat Commun. 2021 Mar 4;12:1448. doi: 10.1038/s41467-021-21759-3 (PMC7933336; doi:10.1038/s41467-021-21759-3)
Supplement: Supplementary file 1 — Supplementary Information [file 41467_2021_21759_MOESM1_ESM.pdf]

Supplementary Information for:

Emerging dominance of summer rainfall driving High Arctic terrestrial-aquatic connectivity

Beel, C.R.[1,2*], **Heslop, J.K.**[1,3*], Orwin, J.F.[1,4], Pope, M.A.[1], Schevers, A.J.[1], Hung, J.K.Y.[1], Lafrenière, M.J.[1], Lamoureux, S.F.[1]

[1]Department of Geography and Planning, Queen's University, Kingston, ON K7L 3N6 Canada
[2]Water Management and Monitoring, Environment and Natural Resources, Government of Northwest Territories, Yellowknife, NT X1A 2L9, Canada
[3]Section 3.7 Geomicrobiology, Helmholtz Centre Potsdam GFZ German Research Centre for Geosciences
[4]Resource Stewardship Division, Alberta Environment and Parks, Government of Alberta, Calgary, AB T2L 2K8, Canada

*These authors contributed equally to this work
Corresponding author: Casey Beel (Casey_Beel@gov.nt.ca)

Here we provide further information on the CBAWO site and our data record (Section 1) and detailed methods (40 references). This document contains an additional 14 supplementary figures and 8 supplementary tables. All data presented in this study are available in the Supplementary Dataset.

Supplementary Note 1

Detailed field site description

This research was conducted at the Cape Bounty Arctic Watershed Observatory (CBAWO), located on the south-central coast of Melville Island in the Canadian High Arctic (74º54'N, 109º35'W, Supplementary Figure 1). This setting is characterized by a polar desert climate (Mean Annual Air Temperature (MAAT; ± 1σ: -14.8 ± 1.3 ºC) with limited annual precipitation and runoff (< 150 mm yr^{-1}) and is underlain by continuous permafrost (~500 m). Mean summer (Jun-Aug; JJA) air temperature is 2.7 ± 2.5 ºC (2003-2019). Extensive deposits of unconsolidated early Holocene marine and Late Glacial sediments drape the underlying sedimentary bedrock (Devonian sandstones and siltstones; Hodgson et al., 1984). Soils are typical cryosols (Soil Classification Working Group, 1998) with a thin organic layer (< 5 cm) and low OC content (1-3 %), developed from largely unaltered parent material (Grewer et al., 2016). Vegetation is characterized as prostrate dwarf-shrub tundra (Walker et al., 2005) with a heterogenous cover composed of wet (16.6 %), mesic (34.7 %), and polar desert (35.1 %) communities depending on slope drainage conditions (Hung and Treitz, 2020).

The CBAWO is comprised of paired watersheds (West and East, unofficial names; WR and ER, respectively); the West watershed is 8.0 km^2 and has instrumented headwater-slope streams (PT: Ptarmigan and GS: Goose; both ~0.2 km^2 watershed area), and the East watershed is 11.6 km^2 (Supplementary Table 1). Both watersheds are non-glacial, and the streams have a seasonal snow-limited flow regime where channel runoff typically begins in early to mid-June, with flow cessation in late-August to early-September during freeze-up (Supplementary Figure 2). Late season rainfall events punctuate baseflow runoff in headwater slope and main watershed streams, with pluvial responses largely controlled by the timing and magnitude of rainfall, as well as antecedent soil moisture conditions (Favaro and Lamoureux, 2015). V-cut notches eroded through a prominent bedrock ridge across both watersheds favour deep snow accumulation in winter (≥ 4 m) and snow dams during nival freshet (Lamoureux et al., 2006). Extensive wind redistribution of winter snow leads to further accumulation in gullies, channels, and concavities, especially in the West watershed (Lewis et al., 2012).

Both the West and East streams have second-order, low-sinuosity channels with short reaches of braided, riffle- and step-pool morphology along their lengths due to variability in channel slope. Surface runoff in the upper East watershed occurs as diffuse flow over vegetated water tracks, hydrologically connecting ponds along the terrestrial-aquatic continuum. Despite the similar relief in both watersheds, the West has more ridges, dissected slopes, and a higher proportion of steeper slopes (Lewis et al., 2012). Water tracks in the West watershed are limited to headwater-slope inputs (e.g., GS; representative of runoff from ca. 50 % of both watersheds area), with the majority of runoff in channelized colluvial streams. Main stream channel bars are dominated by coarse material (55 % cobbles and boulders) draped with patches of fine sediment (45 % silt, clay, and sand) that provide material readily available for remobilization.

Both watersheds are impacted by varying degrees of documented permafrost disturbance. Deep thermal perturbation of the upper permafrost in July 2007, combined with multiple late-season rainfall events, resulted in the formation of 100+ localized active layer detachments (ALDs) that disturbed 2.7 % of the West and 1.2 % of the East watersheds (Lamoureux and Lafrenière, 2017; Supplementary Figure 1). ALDs varied from small, hydrologically (dis)connected patches on slopes to long linear features (> 100 m) that directly coupled with stream networks. Two periods of active massive-ice degradation along channel banks (2008-2010; 2017-ongoing) were observed in a section of the upper West watershed but were absent along any section of the East channel. Analysis of satellite imagery between 1950 (first available image) and 2007 shows that prior to our observed ALDs in 2007-2008 there was no evidence for recent geomorphological disturbance of terrestrial surfaces at the CBAWO (Lamoureux et al., 2014).

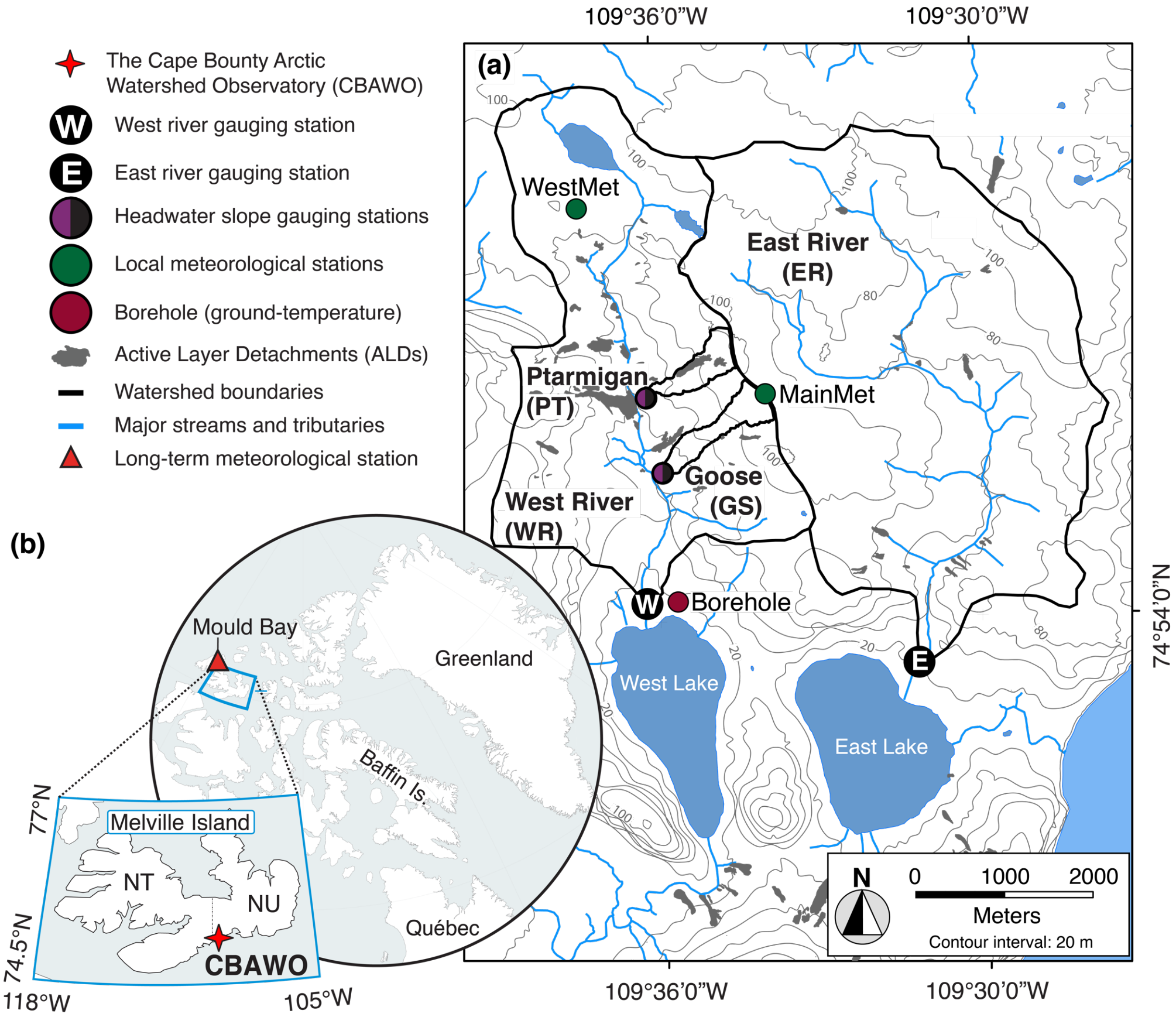

Supplementary Figure 1. The locations of (a) the Cape Bounty Arctic Watershed Observatory (CBAWO) and (b) Melville Island, Nunavut (NU) in the Canadian High Arctic. Boundaries of all studied watersheds are shown, with the locations of stream gauging stations and meteorological stations indicated. ALDs that formed in 2007-2008 are denoted as filled grey polygons that represent mapped disturbance extents on 1 August 2008 (Rudy et al., 2013). Basemaps prepared in ArcGIS *version 10.5* from NTS 1:50 000 map 78F/15 (UTM, WGS 84). Inset map (b) shows the location of Mould Bay, the nearest long-term (1948-2019; 300 km to the west) Environment and Climate Change Canada meteorological site. Maps contain information licensed under the Open Government License – Canada.

Supplementary Table 1. Watershed physiography at the CBAWO.

	Watershed area (km^2)	Mean channel slope	Areal extent of physical permafrost disturbance	Primary stream channel type
Main watersheds				
West	8.0	5.2°	2.7	Colluvial bed
East	11.6	4.5°	1.2	Water track/Colluvial bed
Headwaters				
PT	0.21	3.9°	10.8	Colluvial bed
GS	0.18	3.2°	0	Water track

Supplementary Note 2

Methods

Data consistency and statistics

Instrumentation, datalogger, and analytical method specifications are documented in Supplementary Table 2. For coherence among records and to provide the finest temporal resolution across all years, we reprocessed all variables from raw data into daily means. We used propagation of uncertainty methods to: (1) account for the measurement error associated with instrumentation and analytical methods; and (2) determine how these uncertainties propagate through concentration and flux determinations using the sum of quadrature method (Taylor et al., 1982). To account for interannual variation in hydrometeorological conditions, and since we capture the first day of flow in each year of record, we normalized data to the first day of flow instead of calendar dates for the full data set. All statistical analyses were conducted in Matlab *version R2016a* and *2020a* software. Correlations were calculated using Pearson's r linear correlation coefficients (Supplementary Tables 3-6). Differences between variables: (a) between watersheds and (b) between hydrological periods were determined using a combination of ANOVA analyses (Supplementary Table 7) and two-sample T-tests (Supplementary Figures 9-10). Results from all statistical analyses were considered significant at the $\alpha = 0.05$ level when $p < 0.05$.

Climatology

Climate data were obtained from local and regional meteorological stations proximal to the CBAWO. Local air temperature and rainfall data were collected hourly from the WestMet meteorological station, and barometric pressure data were collected at 10-min intervals at

MainMet (Supplementary Figure 1a). Event-scale rainfall data (2003-2019) from WestMet were used to construct a 72-hr intensity-duration-frequency (IDF) curve for the CBAWO (Gumbel EV1 distribution; Watt et al., 1989) to estimate the recurrence interval for each rainfall event (Supplementary Figure 5; Beel et al., 2018). Additional monthly climate data for Mould Bay, NT (~ 300 km west of the CBAWO; Environment and Climate Change Canada; Supplementary Figure 1b inset) were utilized to evaluate longer-term annual hydrometeorological changes (1948-2019). Given the relatively short duration of available snow and rainfall records at CBAWO and often incomplete records from Mould Bay, evaluation of any changes in the longer-term trend of precipitation beyond our measurement period at the CBAWO is outside the scope of these data.

Active layer depth and growing degree days

Daily JJA active layer depths (Figure 3) were estimated using ground temperature data collected between 2012-2018 from a 7-m borehole (Supplementary Figure 1a). Daily active layer depth estimates were calculated using a linear regression of temperatures recorded at 0.3 m and 1.3 m depth, with the depth at which the temperature was 0 ºC being the assumed thaw boundary. This method assumed linear decreases in ground temperature within the 1.3 m depth range, and actual active layer depths may vary with local soil moisture and ice content conditions; however, values estimated using linear regression (0.0 to 1.20 m during JJA) were consistent with mean seasonal maximum active layer depths manually measured using a probe spatially across the CBAWO (0.7- 1.1 m; Lamoureux and Lafrenière, 2017).

Growing degree days (GDD) represent the cumulative amount of heat above a threshold temperature that supports plant growth and was calculated as:

$$GDD=T_{max}+T_{min}2- T_{base} \text{ (Eq. 1)}$$

where T_{max} and T_{min} are the daily maximum and minimum temperatures respectively and T_{base} is the base temperature representative of the threshold temperature above which plants are productive. Here, we use 5 °C after Carter (1998) and Weijers et al. (2013).

Hydrology

Stream stations located at the outlet of each watershed (WR, ER, PT, GS) measured water-level (stage) and water temperature at 10-min intervals (Supplementary Table 2). Stage measurements were corrected for barometric pressure and converted to discharge (Q) using site-specific annual rating curves developed from: (a) a minimum of 10 velocity-area gauging's under different flow regimes to establish seasonal stage-Q relationships for each main watershed stream ($r^2 = 0.71\text{-}0.99$; Beel et al., 2018); and (b) using calibrated cut-throat flume ratings for headwater-slope streams (Beel et al., 2020). Mean Q uncertainty was calculated to be ± 4-6 % for all streams. Seasonal hydrographs were partitioned into nival (snowmelt), baseflow (low flow), and pluvial (rainfall) hydrological periods based on subjective observed changes in the annual runoff regime (Singh, 1992; Beel et al., 2018; Supplementary Figure 2a). Briefly, the end of the nival period, and start of the baseflow period, was marked by a notable decrease in daily Q and a reduction of the diurnal signal (Supplementary Figure 2a). Pluvial responses were characterized by a rapid increase in Q associated with rainfall inputs. We report seasonal total Q in conventional watershed area-normalized depth (mm) units and instantaneous discharge in volume-time ($m^3\ s^{-1}$) units.

Stream power represents the rate of energy expenditure along a riverbed and banks and was calculated using Equation 2:

$$\Omega = \rho g Q s \qquad \text{(Eq. 2)}$$

where stream power (Ω; Watts or kg $m^{-2}\ s^{-3}$) is the product of water density calculated from measured water temperatures (ρ; kg m^{-3}), acceleration due to gravity (g; 9.8 m s^{-2}), instantaneous discharge (Q; $m^3\ s^{-1}$), and channel slope (s) (Knighton, 1998). We applied a 5 m buffer on either side of the middle of the channel in ArcGIS® to represent the channel area, from which the watershed's average channel slope was calculated (s; Supplementary Table 1).

Dissolved organic and inorganic fluvial measurements

Surface water samples for dissolved organic and inorganic variables were determined from filtered water samples collected between 2006 and 2017. Surface water samples were manually collected in 1 L high-density polyethylene bottles, which were tripled rinsed with

stream water before sample collection. Samples were collected from WR, PT, and GS daily throughout the hydrological season at approximately daily low and high Q (0900 and 1800 h, respectively); at the ER, water samples were collected once every 3-5 days at approximately daily high Q. Samples for both dissolved organic carbon (DOC) and total dissolved nitrogen (TDN) were vacuum-filtered through pre-combusted (400 °C for 4 hr) 0.7-µm GF filters with a glass filtration apparatus. Filtered samples were stored in 45 ml amber EPA vials with Teflon-lined septa following previously published methods (Lewis et al., 2012; Fouche et al., 2017). Note that the pre-combusted 0.7-µm filters were not retained for POC analysis following filtration in the field (Beel et al., 2020). Samples for major ion determination were vacuum filtered through sterile 0.22-µm polyvinylidene difluoride (PVDF) membrane filters with a polysulfone filtration apparatus in the field laboratory and stored in 25 ml scintillation vials with no headspace. Filtered samples were refrigerated in the dark (< 4 °C) until analyses; samples were analyzed within 4-8 weeks of sample collection due to logistical limitations imposed by the remoteness of the CBAWO. We recognize that there was likely some DOC loss in our samples as a result of microbial processing during sample storage, but we are unable to quantify this effect.

DOC and TDN concentrations were measured simultaneously using high-temperature combustion on a Shimadzu TOC-VPCH/TNM system and concentrations of major ions were measured using a Dionex ICS 3000 ion chromatograph at Queen's University, Kingston, Canada. Anions (Cl^-, SO_4^-, NO_3^-) were measured with a gradient elution of 11-40 mM KOH, while cations (Na^+, K^+, Mg^{2+}, Ca^{2+}, NH_4^+) were measured isocratically using 16 mM methane sulfonic acid (MSA) eluent. The detection limits, calculated as three times the standard deviation of the lowest level standard, were less than 0.010 mg L^{-1} for all species, except Ca^{2+} and Mg^{2+}, which had detection limits of 0.053 and 0.022 mg L^{-1}, respectively.

Major ion concentrations were calculated by summing all of the major anions and cations, and bicarbonate (HCO_3^-) concentrations were estimated by charge balance (Lamhonwah et al., 2017). For the ER, daily DOC concentrations were estimated by applying a cubic spline function (*spline*) to the measurements using Matlab *version R2020a* ($p < 0.05$). Failure of refrigerators used to store water samples prior to analysis in 2014 resulted in the loss of a

significant number of filtered water samples. As a result, we are unable to provide an estimate of the DOC_{flux} for ER in 2014. Daily mean major ion concentrations in the ER (2010-2017) were estimated using the relationship between measured major ion and continuous specific electrical conductivity measurements for all available years of data (r = 0.95, $p < 0.05$, $n = 235$) (Supplementary Figure 13).

Daily fluxes (DOC_{flux}, TDN_{flux}, major ion_{flux}) were calculated as the product of total daily Q and daily mean concentrations of DOC, TDN and major ions and summed for each season. Average DOC and TDN uncertainty are calculated at ± 3-5 % (2006-2010) and ± 5-8 % (2012-2017). Average major ion uncertainty is calculated at ± 4-6 %. Total uncertainties for annual DOC_{flux} and TDN_{flux} were estimated to be ± 6-10 % (2006-2010) and ± 7-12 % (2012-2017). Total uncertainties for annual major ion_{flux} were estimated to be ± 5-9 %, respectively.

DOM optical measurements

Optical properties of chromophoric dissolved organic matter (CDOM) and fluorescent dissolved organic matter (FDOM) were determined using a Horiba Aqualog from DOC samples collected between 2012 and 2017. Both UV-visible absorbance (CDOM) and fluorescence (FDOM) were measured in a 1 cm quartz cuvette at room temperature. UV-visible absorbance was measured from 240 to 600 nm in 3 nm increments, then linearly interpolated to 1 nm increments. Excitation values were measured from 240 to 600 nm in 3 nm increments and corresponding emission wavelengths were measured from 214.16 to 621.03 nm at 3.15 nm intervals. CDOM measurements were referenced against DI water blanks and normalized using a 1 ppm quinine sulfate standard. FDOM excitation-emission matrix (EEM) files were: (1) corrected for blanks and inner filter effects (McKnight et al. 2001); (2) first- and second-order Rayleigh scatter effects were removed using the manufacturer's correction procedure; and (3) normalized using a 1 ppm quinine sulfate standard.

CDOM data were used to calculate specific UV absorbance at 254 nm ($SUVA_{254}$), a parameter that is commonly used as an index of DOM aromaticity, by dividing the measured absorbance at a wavelength of 254 nm by the sample's DOC concentration (L mgC^{-1} m^{-1}; Weishaar et al., 2003). We then converted measured absorbance values (A) to Naperian units (α) using Equation 3:

$$\alpha(\lambda) = 2.303A(\lambda)/l \qquad \text{(Eq. 3)}$$

where A(λ) is the measured absorbance at wavelength λ and l is the cell path length in meters (Green and Blough, 1994). We report absorption coefficients, in Naperian units, at wavelengths of 254 nm (α_{254}) and 350 nm (α_{350}). α_{254} and α_{350} have been previously shown to correlate with allochthonous-dominated DOM in riverine systems (Spencer et al., 2012). Further, α_{350} has been previously shown to strongly correlate with lignin phenol concentrations (Spencer et al., 2010) and are indicative of terrigenous DOM in riverine systems (Mann et al., 2016; Spencer et al., 2012). Spectral slope was calculated between 275-295 nm and 350-400 nm by fitting Equation 4:

$$\alpha_g(\lambda) = \alpha_g(\lambda_{ref})e^{-S(\lambda- \lambda ref)} \qquad \text{(Eq. 4)}$$

where $\alpha_g(\lambda)$ is the Naparian absorption coefficient at wavelength λ, λ_{ref} is a reference wavelength, and S is the slope fitting parameter (Helms et al., 2008). Spectral slope ratio (S_R; $S_{275-295}$:$S_{350-400}$) have been shown to correlate with average DOM molecular weight (Helms et al., 2008).

Using the FDOM data, we calculated the Fluorescence Index (FI), a measure of the relative contribution of terrestrial and microbial DOM sources, as the ratio of measured intensities at ex370/em470 and ex370/em520 (Cory et al., 2010). Humification index (HIX), an indicator of the degree of DOM humification, was calculated by dividing the area under ex254/em435-480 by the summed area under ex254/em300-345 and ex254/em435-480 (Ohno, 2002). We calculated the freshness index (β:α), an indicator of recently-produced DOM, as the ratio of intensity at ex310/em380 divided by the maximum intensity between ex310/em420-435 (Wilson and Xenopoulos, 2009). Additionally, the biological index (BIX), an indicator of

autochthonous-origin DOM, was determined at the ratio of intensity at ex310/em380 divided by intensity at ex310/em430 (Huguet at al., 2009).

Particulate organic and inorganic fluvial measurements

Concentrations of suspended sediment (SSC) and particulate organic carbon (POC) were determined from water samples collected every 3 hr at main watershed stream stations (2003-2017) and concurrently with dissolved samples in headwater-slope streams (2007-2017). All samples were volumetrically filtered through non-combusted, pre-weighed 1-µm glass fiber (GF) filters, folded to retain sediment on the filter and stored in individual polyethylene bags for SSC and POC quantification. Although pre-combusted 0.7-µm GF filters were not available for POC analysis in this study, extensive testing of non-combusted 1-µm GF filtration versus pre-combusted filters with 0.7-µm pore sizes showed negligible differences in concentrations of POC from these watersheds (Beel et al., 2020), similar to observations from the Mackenzie River (Emmerton et al., 2008; Gareis and Lesack, 2017).

In the laboratory, the GF filters were freeze-dried then weighed twice to determine SSCs. Average SSC uncertainty is ± 4 % (> 10 mg L^{-1}) and ± 10 % (< 10 mg L^{-1}). To determine POC concentrations, filters were acid-fumed with 50 ml of 6 % trace metal grade sulfurous acid (H_2SO_3) for 20-hr and oven-dried overnight at 50 °C (Kennedy et al., 2005; Komada et al., 2008). After fumigation, filters were subsampled into quarters, weighed individually, and pelletized in foil cups. Sample pellets were analyzed for organic carbon (OC) content using a LECO TruSpec CN elemental analyzer (Beel et al., 2020). Process blanks and LECO certified reference materials (LOT 1000: 10.8 ± 0.26 % C, 0.86 ± 0.03 % N) were run at the beginning and throughout every run to ensure consistency and determine instrument accuracy and stability (Beel et al., 2020). POC mass was calculated as the product of the sediment mass retained on the filter and the proportion of sediment composed of C (wt%), which was divided by filtered water volumes to determine POC concentrations (mg L^{-1}).

SSC and POC were strongly linearly correlated in both headwater slope (r^2 = 0.96-0.99; Beel et al., 2020) and main watershed streams (r^2 = 0.8-1.0; Supplementary Figure 14). In headwater-slope streams, the proportion of POC in SSC ranged from 0.9 to 2.1 %, with POC

averaging 1.4 % of the SSC in both PT and GS (Beel et al., 2020). In the main watershed streams, the proportion of POC in SSC ranged from 0.9 to 3.5 %, with POC averaging 2.0 % and 1.8 % of the SS in the WR and ER, respectively (Supplementary Data).

The slopes of the correlation between SSC and POC varied between years suggesting the need for annual, site-specific relationships to be established (Supplementary Figure 14). Average POC uncertainty was ± 6 % (POC > 10 mg L^{-1}) and ± 13 % (POC ≤ 10 mg L^{-1}). Daily fluxes (kg; SS_{flux}, POC_{flux}) were calculated with the product of total daily Q and daily mean SSC and POC and summed for each season. Total uncertainties for annual SS_{flux} and POC_{flux} were estimated to be ± 4.5-10 % and ± 8-15 %, respectively.

Supplementary References

Beel, C.R., Lamoureux, S.F., Orwin, J.F. Fluvial response to a period of hydrometeorological change and landscape disturbance in the Canadian High Arctic. *Geophys. Res. Lett.,* 45, https://doi.org/10.1029/2018GL079660 (2018).

Beel, C.R., *et al.* Differential impact of thermal and physical permafrost disturbance on High Arctic dissolved and particulate fluvial fluxes. *Sci. Report.*, 10, 11836. https://doi.org/10.1038/s41598-020-68824-3 (2020).

Carter, T. Changes in the thermal growing season in Nordic countries during the past century and prospects for the future. *Agricultural and Food Science in Finland*, 7, 161–179. https://doi.org/10.23986/afsci.72857 (1998).

Cory, R.M., *et al.* Effect of instrument-specific response on the analysis of fulvic acid fluorescence spectra. *Limnol. Oceanogr.-Meth.* **8**, 67-78 (2010).

Emmerton, C.A., Lesack, L.F.W., & Vincent, W.F. Mackenzie River nutrient delivery to the Arctic Ocean and effects of the Mackenzie Delta during open water conditions. *Global Biogeochem. Cycles*, 22, GB1024, https://doi.org/10.1029/2006GB002856 (2008).

Favaro, E.A., & Lamoureux, S.F. Antecedent controls on rainfall runoff response and sediment transport in a High Arctic catchment. Geogr. Ann. Phys. Geogr., 96(4), 433-446. https://doi.org/10.1111/geoa.12063 (2014).

Fouché, J., Lafrenière, M.J., Rutherford, K., & Lamoureux, S.F. Seasonal hydrology and permafrost disturbance impacts on dissolved organic matter composition in High Arctic headwater catchments. *Arctic Sci.*, 3, 378-405, https://doi.org/10.1139/as-2016-0031 (2017).

Gareis, J.A.L., & Lesack, L.F.W. Fluxes of particulates and nutrients during hydrologically defined seasonal periods in an ice-affect great Arctic river, the Mackenzie. *Water Resour. Res.*, 53, 6109-6132, https://doi.org/10.1002/2017WR020623 (2017).

Green, S.A. and Blough, N.V. Optical absorption and fluorescence properties of chromophoric dissolved organic matter in natural waters. *Limn. Oceanogr.* **39**, 1903-1916 (1994).

Grewer, D.M., Lafrenière, M.J., Lamoureux, S.F., Simpson, M.J. Redistribution of soil organic matter by permafrost disturbance in the Canadian High Arctic. *Biogeochemistry*, 128(3), 397-415, https://doi.org/10.1007/s10533-016-0215-7 (2016).

Government of Canada – Environment and Natural Resources. *Historical Climate Data* (www.climat.meteo.gc.ca, 2019).

Helms, J.R., *et al.* Absorption spectral slopes and slope ratios as indicators of molecular weight, source, and photobleaching of chromophoric dissolved organic matter. *Limnol. Oceanogr.* 53, 955-969. https://doi.org/10.4319/lo.2008.53.3.0955 (2008).

Hodgson, D.A., Vincent, J.-S., Fyles, J.G. Quaternary geology of central Melville Island, Northwest Territories. *Geological Survey of Canada*, Paper 83-16, https://doi.org/10.4095/119784 (1984).

Huguet, A., *et al.* Properties of fluorescent dissolved organic matter in the Gironde Estuary. *Org. Geochem.*, 40(6), 706-719. https://doi.org/10.1016/j.orggeochem.2009.03.002 (2009).

Hung, J.K.Y., Treitz, P. Environmental land-cover classification for integrated watershed studies: Cape Bounty, Melville Island, Nunavut. *Arctic Sci.*, *early version*, 1-19. https://doi.org/10.1139/as-2019-0029 (2020).

Kennedy, P., Kennedy, H., & Papadimitriou, S. The effect of acidification on the determination of organic carbon, total nitrogen and their stable isotopic composition in algae and marine sediment. *Rapid Commun. Mass Spectrom.*, 19, 1063-1068, https://doi.org/10.1002/rcm.1889 (2005).

Komada, T., Anderson, M.R., & Dorfmeier, C.L. Carbonate removal from coastal sediments for the determination of organic carbon and its isotopic signatures, d^{13}C and D^{14}C: comparison of fumigation and direct acidification by hydrochloric acid. *Limnol. Oceanogr.: Methods*, 6, 254-262, https://doi.org/10.4319/lom.2008.6.254 (2008).

Knighton, D. *Fluvial forms & processes*. (Hodder Arnold, 1998).

Lamhonwah, D., Lafrenière, M.J., Lamoureux, S.F., & Wolfe, B.B. Multi-year impacts of permafrost disturbance and thermal perturbation on High Arctic stream chemistry. *Arctic Sci.*, 3, 254-276, https://doi.org/10.1139/as-2016-0024 (2017).

Lamoureux, S.F., *et al.*, An incidence of multi-year sediment storage on channel snowpack in the Canadian High Arctic. *Arctic*, 59(4), 381-390, https://doi.org/10.14430/arctic287 (2006).

Lamoureux, S.F., Lafrenière, M.J. More than just snowmelt: integrated watershed science for changing climate and permafrost at the Cape Bounty Arctic Watershed Observatory. *WIREs Water*, 5(1), e1255, https://doi.org/10.1002/wat2.1255 (2017).

Lewis, T., Lafrenière, M.J., Lamoureux, S.F. Hydrochemical and sedimentary responses of paired High Arctic watersheds to unusual climate and permafrost change, Cape Bounty, Melville Island, Canada. *Hydrol. Process.*, 26, 2003-2018, https://doi.org/10.1002/hyp.8335 (2012).

Mann, P.J., et al. Pan-Arctic trends in terrestrial dissolved organic matter from optical measurements. Front. Earth. Sci., 4:25. https://doi.org/10.3389/feart.2016.00025 (2016).

McKnight, D.M., *et al.*, Spectrofluorometric characterization of dissolved organic matter for indication of precursor organic material and aromaticity. *Limno. Ocean.*, 46(1), 38-48. https://doi.org/10.4319/lo.2001.46.1.0038 (2001).

Ohno, T., et al. Ultrahigh Resolution Mass Spectrometry and Indicator Species Analysis to Identify Marker Components of Soil- and Plant Biomass-Derived Organic Matter Fractions. Environmental Science & Technology 44, 8594–8600 (2010).

Rudy. A.C.A., Lamoureux, S.F., Treitz, P., Collingwood, A. Identifying permafrost slope disturbance using multi-temporal optical satellite images and change detection techniques. *Cold Reg. Sci. Technol.*, **88**, 37-49, https://doi.org/10.1016/j.coldregions.2012.12.008 (2013).

Singh, V. *Elementary Hydrology*. (Prentice Hall, 1992).

Soil Classification Working Group. The Canadian System of Soil Classification (3rd Ed.) *Agriculture and Agri-Food Canada Publication*, 1646 (Revised, 1998). http://sis.agr.gc.ca/cansis/publications/manuals/1998-cssc-ed3/index.html

Spencer, R.G.M., et al. Comparison of XAD with other dissolved lignin isolation techniques and a compilation of analytical improvements for the analysis of lignin in aquatic settings. *Org. Geochem.*, 41, 445-453. https://doi.org/10.1016/j.orggeochem.2010.02.004 (2010).

Spencer, R.G.M., *et al.*, Dissolved organic carbon and chromophoric dissolved organic matter properties of rivers in the USA. *J. Geophys. Res.*, 117, G03001. https://doi.org/10.1029/2011JG001928 (2012).

Taylor, J.R. *An introduction to error analysis: the study of uncertainties in physical measurements*. (University Science Books, 1982).

Walker, D.A. *et al.*, The Circumpolar Arctic vegetation map. *J. Veg. Sci.*, 16, 267-282. https://doi.org/10.1111/j.1654-1103.2005.tb02365.x (2005).

Watt, W.E., Lathem, K.W., Neill, C.R., Richards, T.L., & Rousselle, J. *Hydrology of Floods in Canada: A Guide to Planning and Design* (National Research Council of Canada, 1989).

Weijers, S., Wagner-Cremer, F., Sass-Klaassen, U., Broekman, R., Rozema, J. Reconstructing High Arctic growing season intensity from shoot length growth of a dwarf shrub. *Holocene*, 23(5), 721–731. https://doi.org/10.1177/0959683612470178 (2013).

Weishaar, JL, et al. Evaluation of specific ultraviolet absorbance as an indicator of the chemical composition and reactivity of dissolved organic carbon. Environ. Sci. Technol. 37, 4702-4708 (2003).

Wilson, H.F., Xenopoulos, M.A. Effects of agricultural land use on the composition of fluvial dissolved organic matter. *Nat. Geosci.*, 2, 37– 41. https://doi.org/10.1038/NGEO391 (2009).

Supplementary Figures

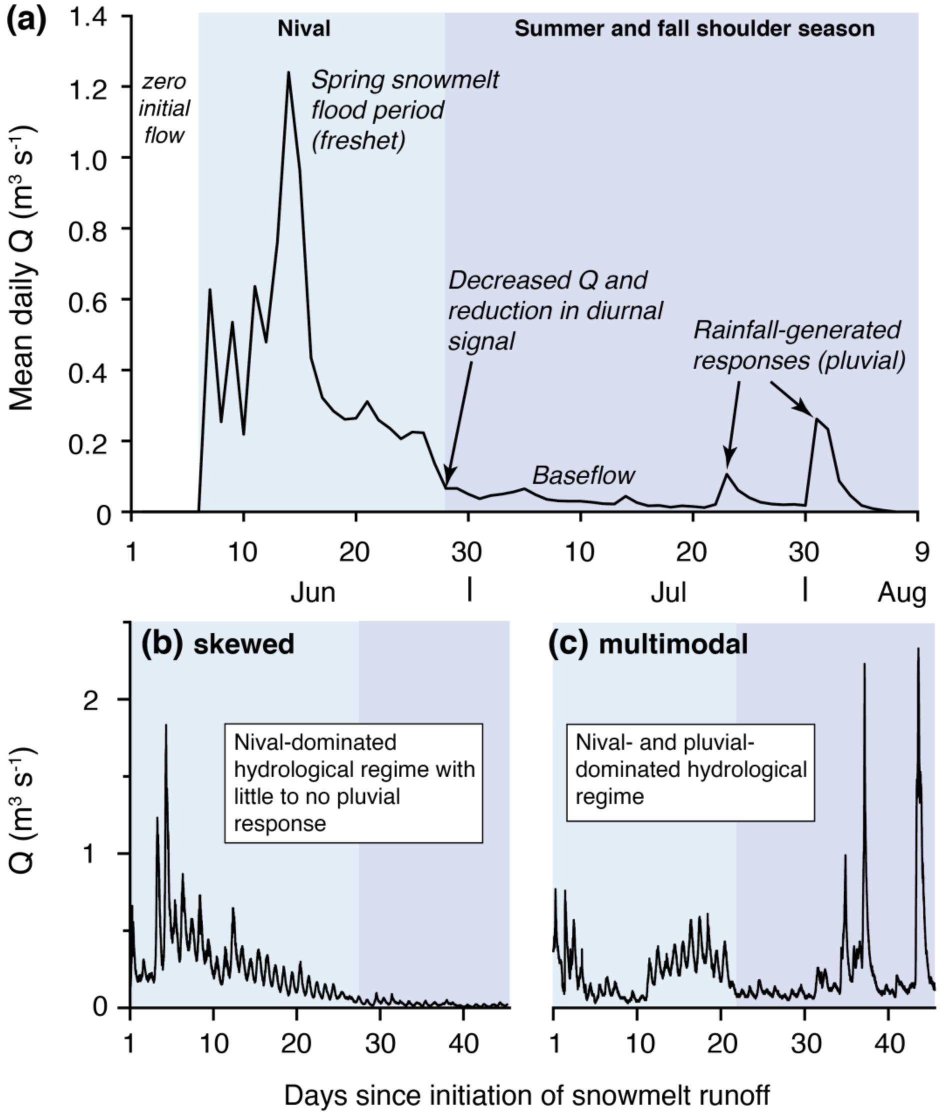

Supplementary Figure 2. Typical nival-dominated hydrological regime for a small High Arctic stream, West river, CBAWO. (a) Mean daily discharge (Q) for the 2016 thaw season. The snowmelt (nival) period begins with a rapid rise in Q from zero initial flow to peak annual Q within a few days. As the watershed snowpack is depleted, Q rapdily declines to baseflow characterized by a reduction in peak Q and diurnal variability. Baseflow periods are often punctuated by rainfall-generated (pluvial) Q responses during the summer and fall shoulder season. In years with little to no pluuvial response in the summer/fall shoulder season (b) stream hydrographs are skewed or classfied as nival-dominated hydrology. In years with proportionately more pluvial runoff (a and c), the timing of seasonal stream power shifted from a skewed (b) to a multimodal hydrological regime. This shift in fluvial energy to later in the thaw season elevated the potential for lateral and longitudinal material fluxes along the terrestrial-aqautic continuum due to increased hydrological connectivity.

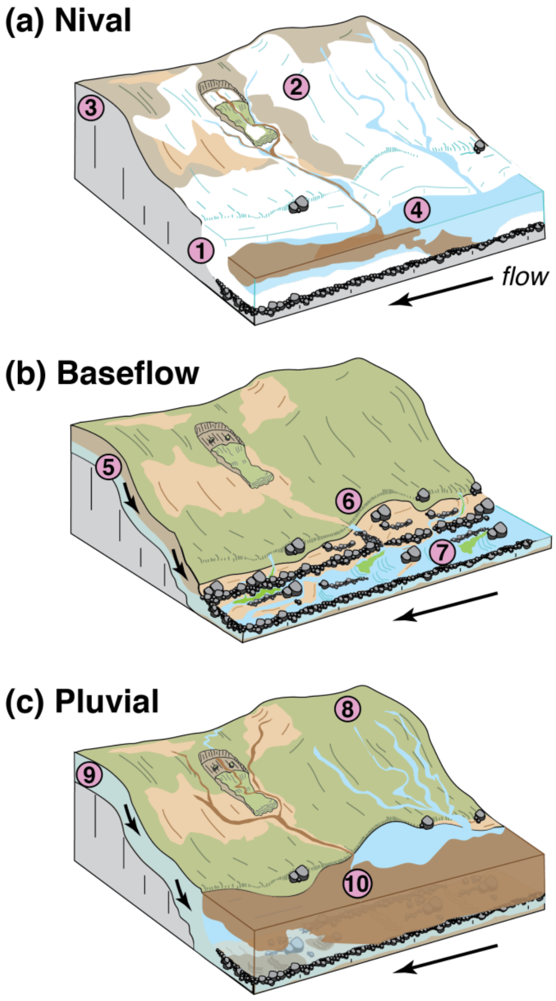

Supplementary Figure 3. Differences in the terrestrial-to-aquatic connectivity of High Arctic watersheds underlain by continuous permafrost during different hydrological periods: (a) nival (snowmelt); (b) baseflow; and (c) pluvial (rainfall). The nival period is characterized by: (1) snow-channelized flow largely in isolation from the channel bed and (2) terrestrial surfaces; (3) shallow active layer depths (0-5 cm); and (4) mobilization and transfer of shallow terrestrial and channel bed material. Baseflow periods are defined by: (5) increased subsurface hydrological coupling as the active layer deepens; (6) Surface flow in headwater slope streams ceases following snow exhaustion, transitioning to a subsurface flow regime with limited coupling between terrestrial surfaces and stream channels; and (7) increased in-stream biological activity and processing of modern terrigenous DOM. Summer rainfall (8) couples the terrestrial-to-aquatic cascade at a time when (9) the seasonal active layer is deepening ($\leq 1.0 \pm 0.2$ m) and vegetation is growing, increasing interactions between surface and subsurface terrestrial environments and stream networks; (10) Rainfall events and subsequent pluvial runoff had to increase by an order of magnitude to effectively couple terrestrial-aquatic pathways along the full watershed continuum for particulate terrigenous materials.

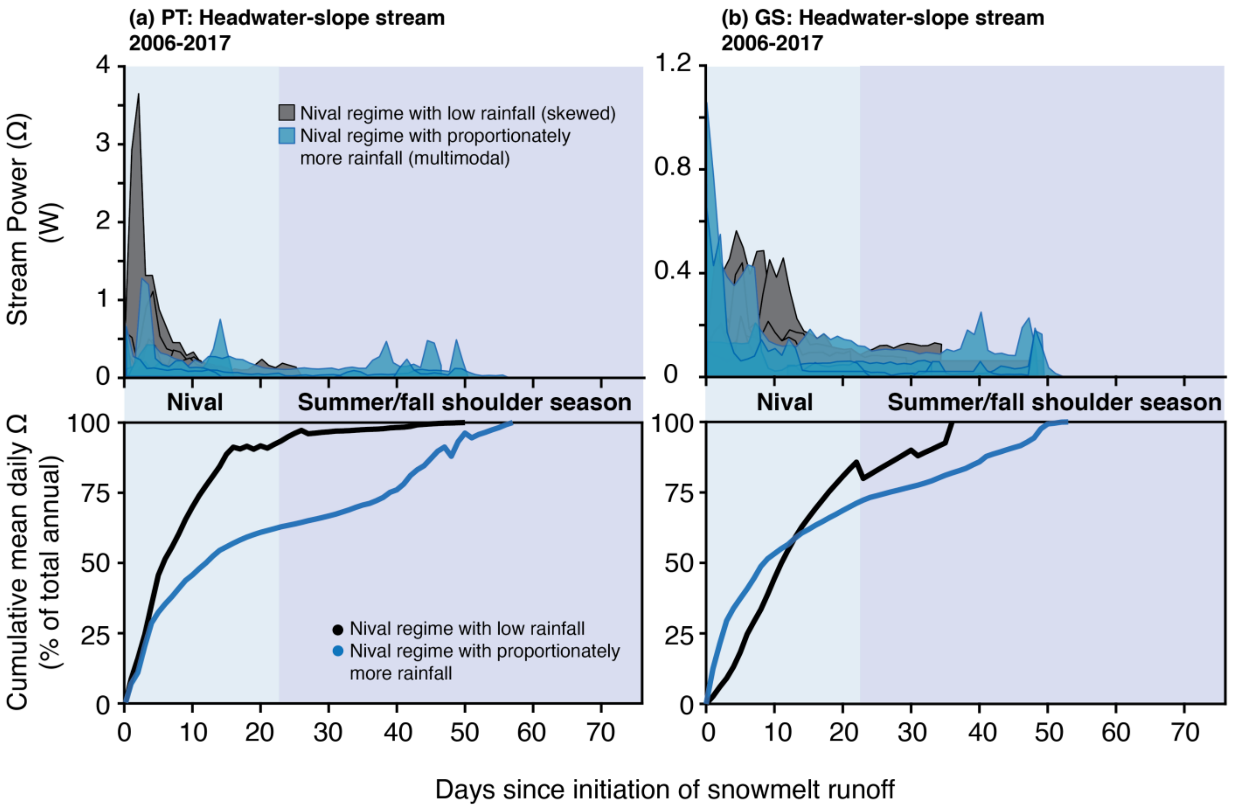

Supplementary Figure 4. Daily stream power records (top panels) and the cumulative mean daily stream power (% of the total annual stream power; bottom panels) for the headwater slope streams (0.2 km^2): (a) Ptarmigan (PT; 2006-2017) and (b) Goose (GS; 2006-2017); and main watershed streams (10 km^2) at the CBAWO: (c) West river (WR; 2003-2017) and (d) East river (ER; 2003-2017). Note in nival-dominated, low rainfall years (n = 8; black line), ≥ 50 % of the annual available stream power is expended within the first ten days of runoff and 80 ± 7 % of annual available stream power is expended during the full nival period (20 days). In warmer years with proportionately more rainfall (n = 4; blue line) 25-40 % of the annual available stream power is expended later in the thaw season. Low energy baseflow runoff expends < 5 % of the annual available stream power. Data from this figure are in Figure 1.

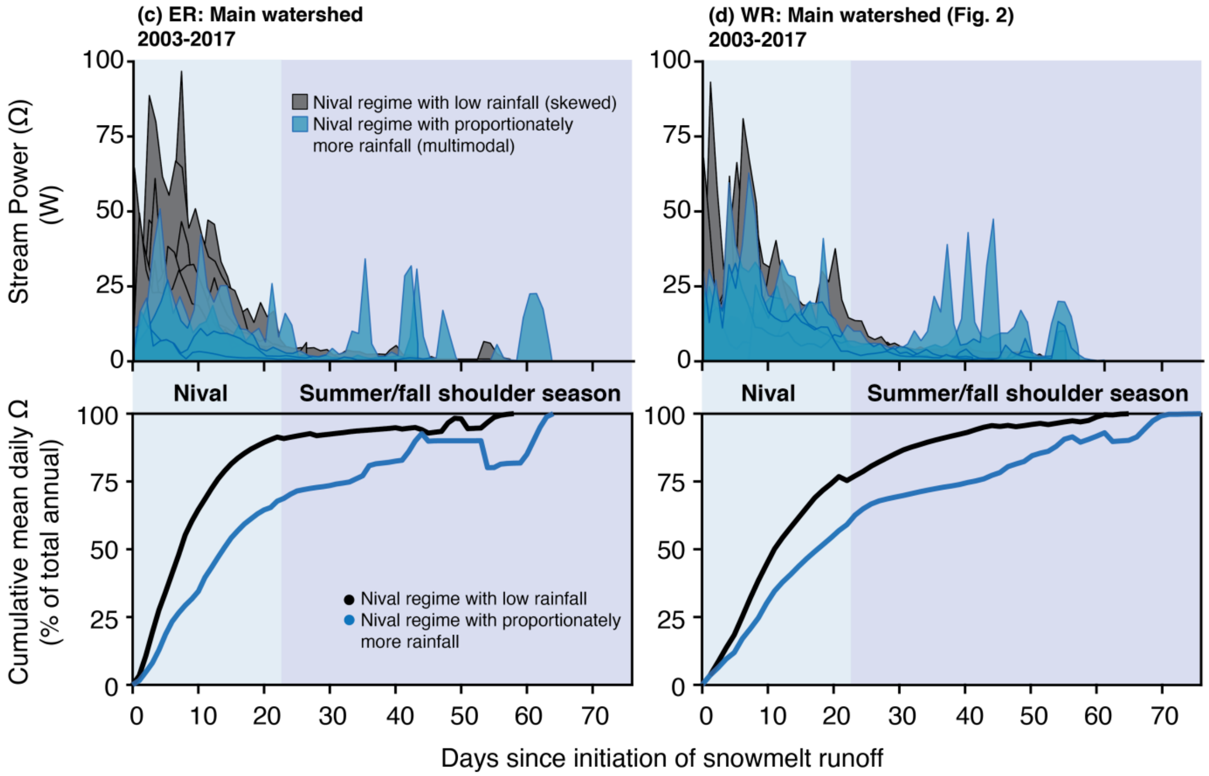

Supplementary Figure 4 cont. Daily stream power records (top panels) and the cumulative mean daily stream power (% of the total annual stream power; bottom panels) for the headwater slope streams (0.2 km^2): (a) Ptarmigan (PT; 2006-2017) and (b) Goose (GS; 2006-2017); and main watershed streams (10 km^2) at the CBAWO: (c) West river (WR; 2003-2017) and (d) East river (ER; 2003-2017). Note in nival-dominated, low rainfall years (n = 10; black line), ≥ 50 % of the annual available stream power is expended within the first ten days of runoff and 80 ± 7 % of annual available stream power is expended during the full nival period (20 days). In warmer years with proportionately more rainfall (n = 4; blue line) 25-40 % of the annual available stream power is expended later in the thaw season. Low energy baseflow runoff expends < 5 % of the annual available stream power. Data from this figure are in Figure 1.

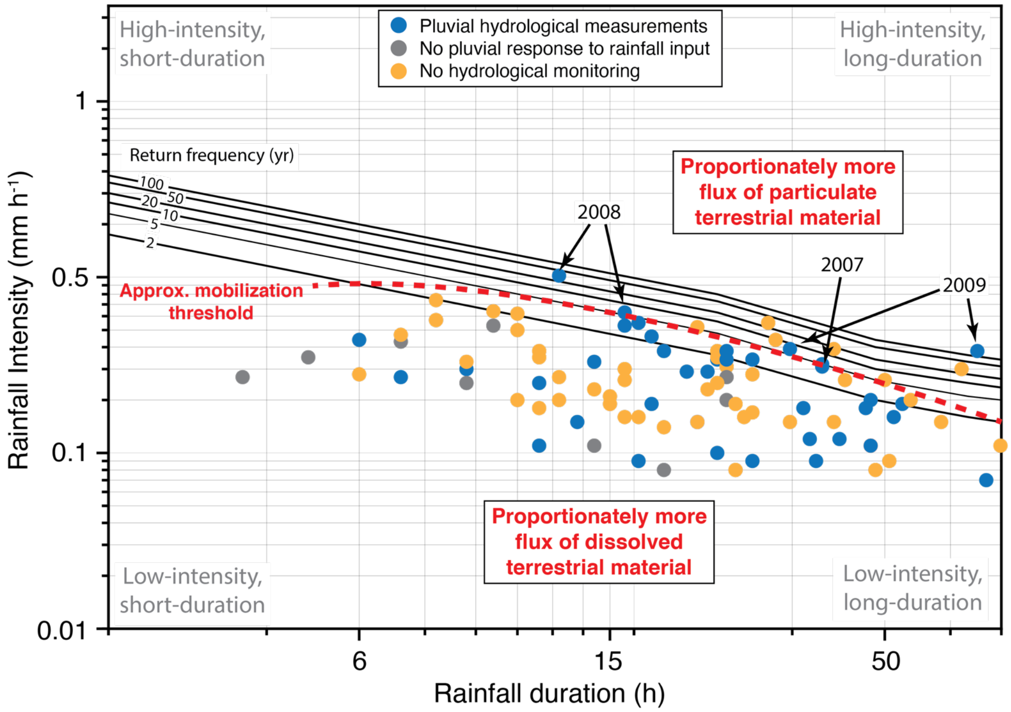

Supplementary Figure 5. The 72-h intensity-duration-frequency (IDF) curve for the CBAWO overlain with all measured rainfall events between 2003-2019 (n = 100). We have pluvial records for 39 of the 100 rainfall events (39 %; blue dots), including the highest magnitude rainfall event on record (2009; 100-year return frequency or 1% chance of exceedance in any given year). Maximum stream power during the 2007 and 2009 rainfall events exceeded that of the nival period. Although we captured significant High Arctic pluvial responses, our understanding remains limited by hydrological monitoring failing to capture the majority of late summer/fall (Aug-Sep) rainfall events (61 %; yellow dots). Note that not all rainfall events resulted in measurable pluvial responses at these watershed scales (grey dots), and high-intensity short-duration rainfall events in 2008 did not produce significant pluvial response due to antecedent conditions. The red dashed line indicates the approximate rainfall magnitude pluvial mobilization threshold: low-to medium-magnitude rainfall events (below the line) produce enough energy to couple the watershed-scale terrestrial-aquatic cascade for dissolved material, while higher magnitude rainfall events (above the line) account for the majority of particulate flux and connectivity from hillslope sediment sources to watershed outlets.

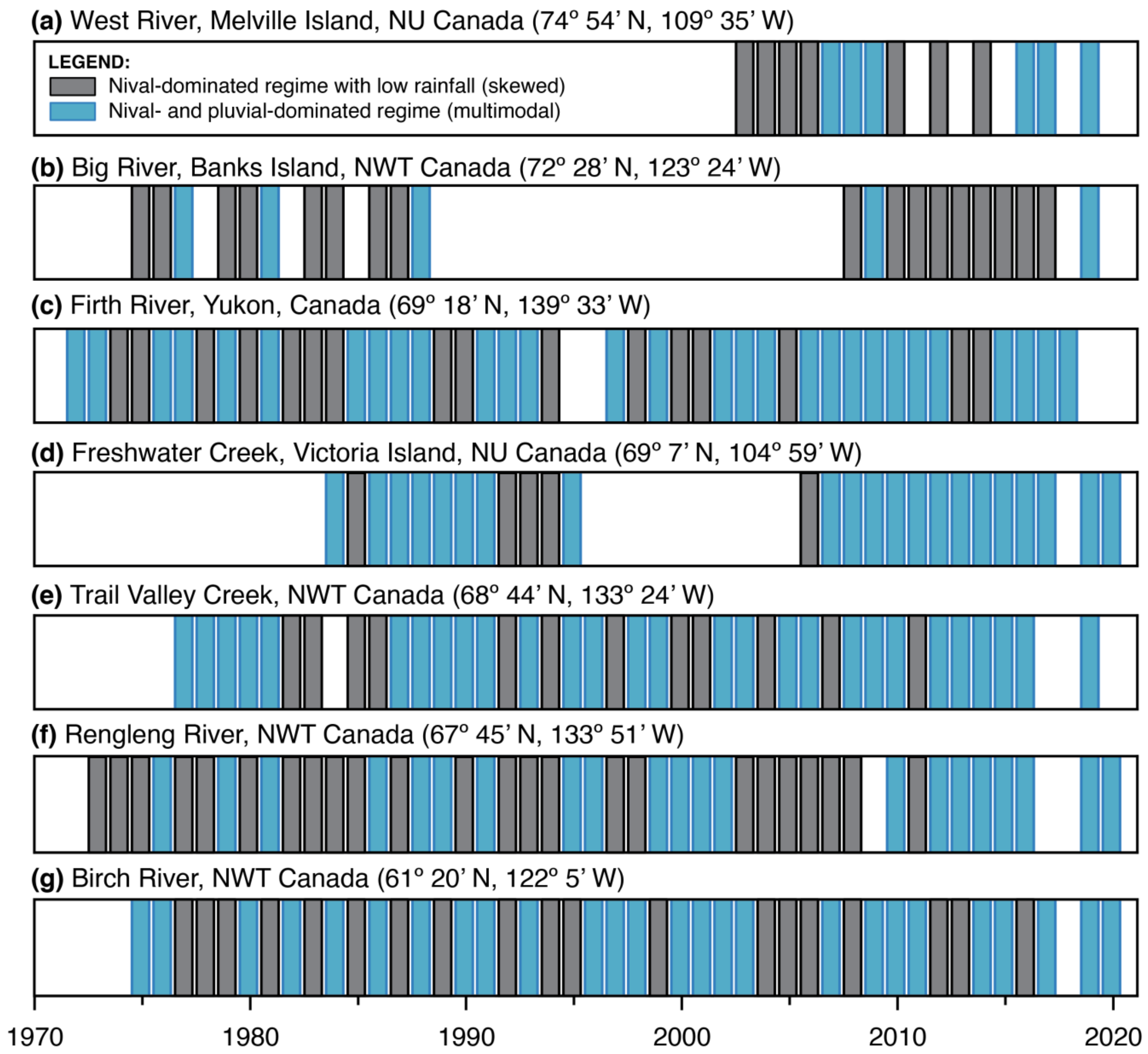

Supplementary Figure 6. Summary of annual hydrological regimes along a northern Canadian latitudinal gradient (and varying in watershed size); from north to south: (a) West River, CBAWO (74 °N); (b) Big River, Banks Island NT (72 °N); (c) Firth River, YT (69 °N); (d) Freshwater Creek, Victoria Island, NU (69 °N); (e) Trail Valley Creek, NT (68 °N); (f) Rengleng River, NT (67 °N); and (g) Birch River, NT (61 °N). Note that the observed changes at CBAWO are ubiquitous across the Canadian High and Low Arctic though watersheds in the Low Arctic received proportionately more rainfall in general. Annual hydrology grouped into skewed (nival-dominated) and multimodal (nival- and pluvial-dominated) regimes based on subjective observed changes outlined in Supplementary Figure 2b & 2c. Data sourced from the Global Runoff Data Center (GRDC, 2020; www.portal.grdc.bafg.de).

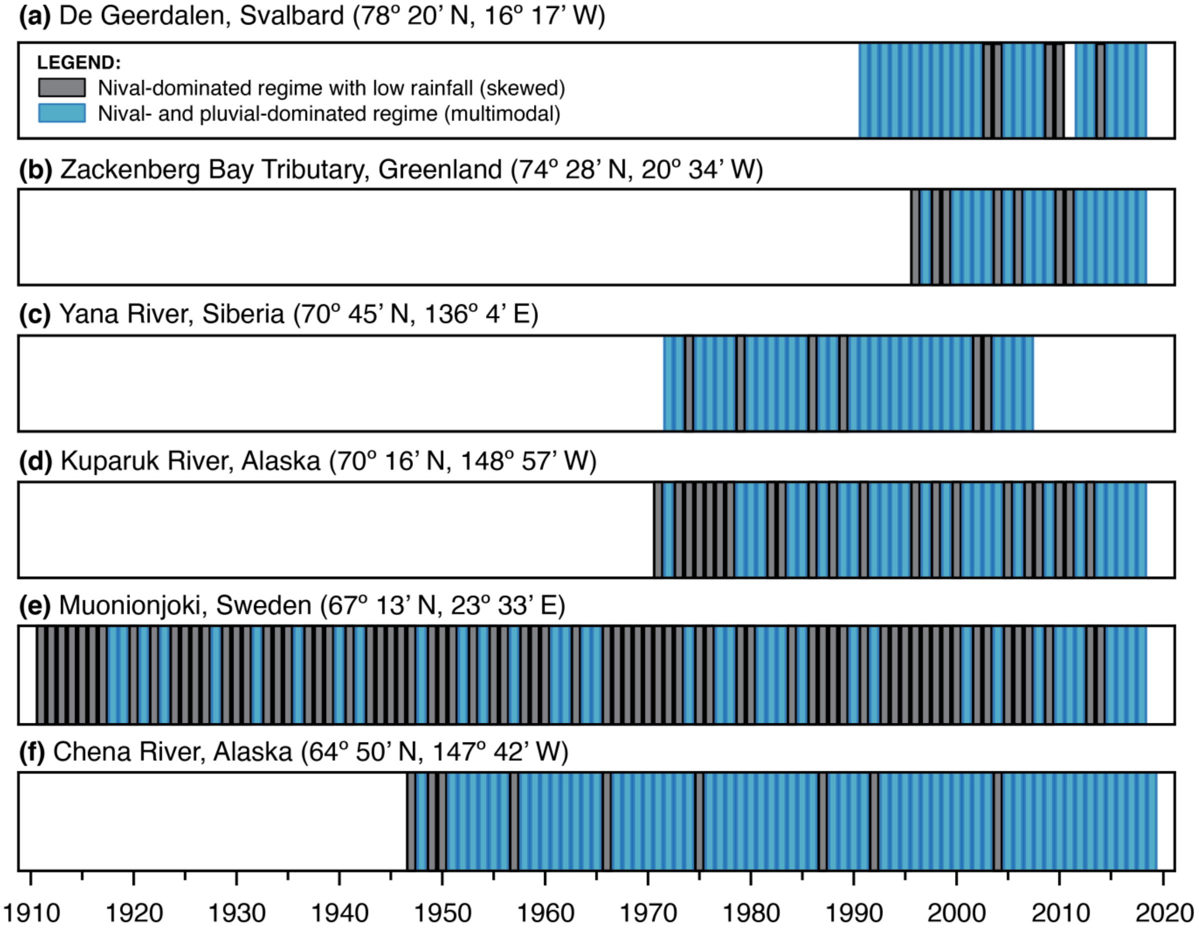

Supplementary Figure 7. Summary of annual hydrological regimes across the circum-Arctic; (a) De Geerdalen, Svalbard; (b) Zackenberg Bay Tributary, eastern Greenland; (c) Yana River, Siberia; (d) Kuparuk River, Alaska; (e) Muonionjoki, Sweden; and (f) Chena River, Alaska. Note that the observed changes at CBAWO are ubiquitous across the circum-Arctic. Annual hydrology grouped into skewed (nival-dominated) and multimodal (nival- and pluvial-dominated) regimes based on subjective observed changes outlined in Supplementary Figure 2b & 2c. Data sourced from the Global Runoff Data Center (GRDC, 2020; www.portal.grdc.bafg.de).

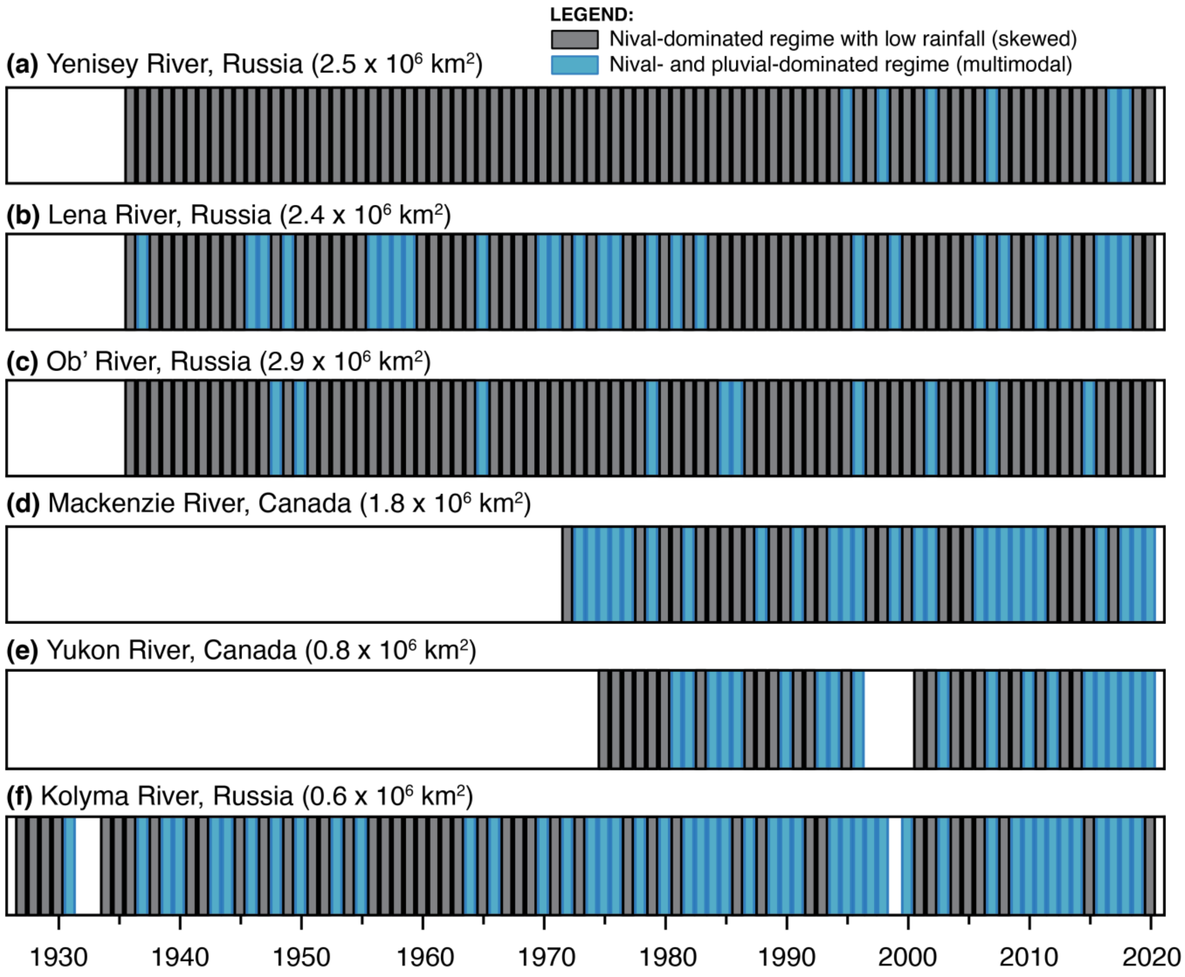

Supplementary Figure 8. Summary of annual hydrological regimes from the six largest Arctic rivers (0.65-2.95 million km^2): (i) Yenisey River; (ii) Lena River; (iii) Ob' River; (iv) Mackenzie River; (v) Yukon River; and (vi) Kolyma River. Note that observed changes in small arctic watersheds ($\leq$ 225,000 km^2) are observed in the six largest Arctic rivers, though they received proportionately more rainfall in general. Annual hydrology grouped into skewed (nival-dominated) and multimodal (nival- and pluvial-dominated) regimes based on subjective observed changes outlined in Supplementary Figure 2b & 2c.

Data sourced from: Shiklomanov, A.I., R.M. Holmes, J.W. McClelland, S.E. Tank, and R.G.M. Spencer. 2020. Arctic Great Rivers Observatory. Discharge Dataset, Version 20201209.

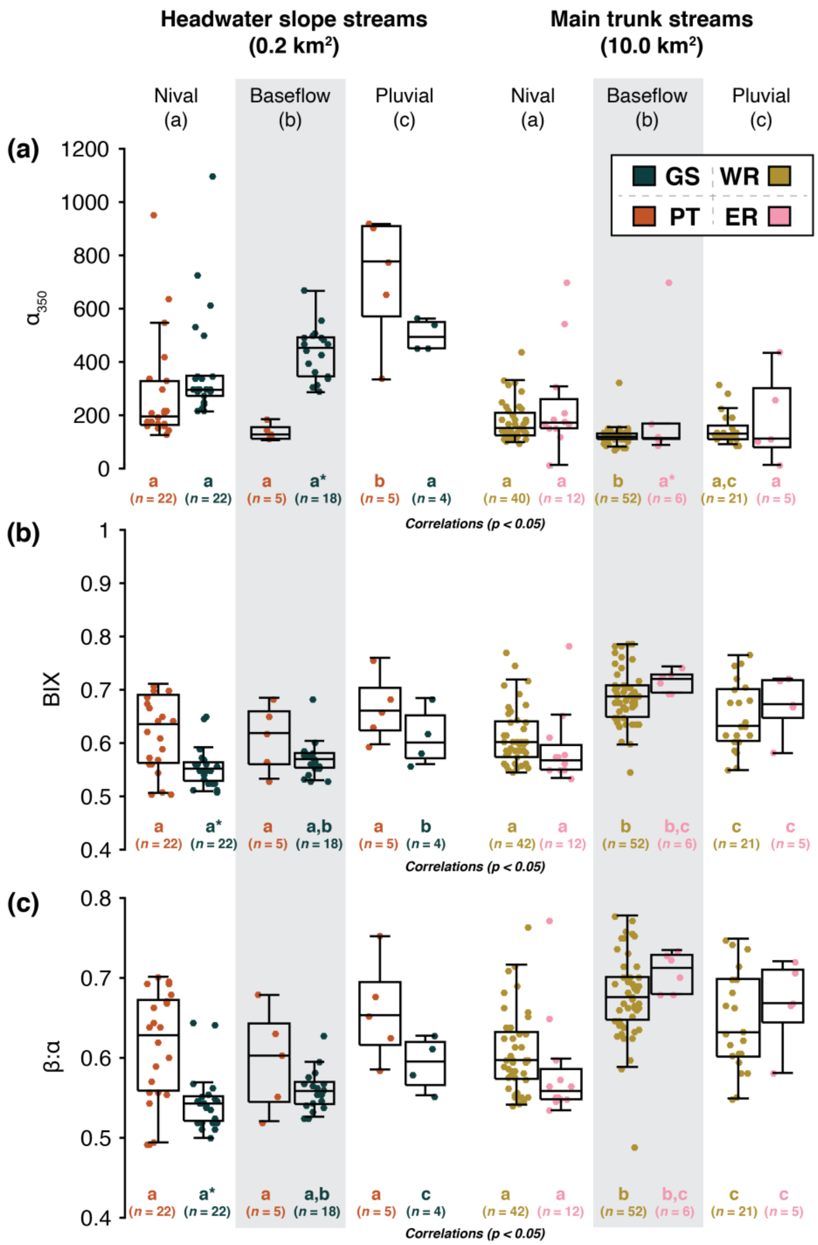

Supplementary Figure 9. Box plots showing the DOM optical properties: α_{350} (a), an indicator of terrigenous vegetation-derived DOM; BIX (b), an indicator of autochthonous DOM; and the freshness index (β:α; c), an indicator of recently-produced DOM. Each box plot shows the median (white line), interquartile range (colored boxes), upper and lower fences (black line "whiskers"; calculated as the upper/lower interquartile range limit ± 1.5 times the interquartile range), and potential outlier observations (colored plus symbols). Statistically different differences between hydrological periods (two-sample t-test; $p < 0.05$) are noted using letters below each box plot (e.g. if the nival and baseflow period both have "a"s they are not significantly different, while a change of letter between periods, or unique letter combinations "a,b" vs. "c" are significantly different); asterisks signify significant ($p<0.05$) differences between the two watersheds for the same hydrological period. The number of observations used to calculate each box plot are noted beneath. All data are available in the Supplementary Data.

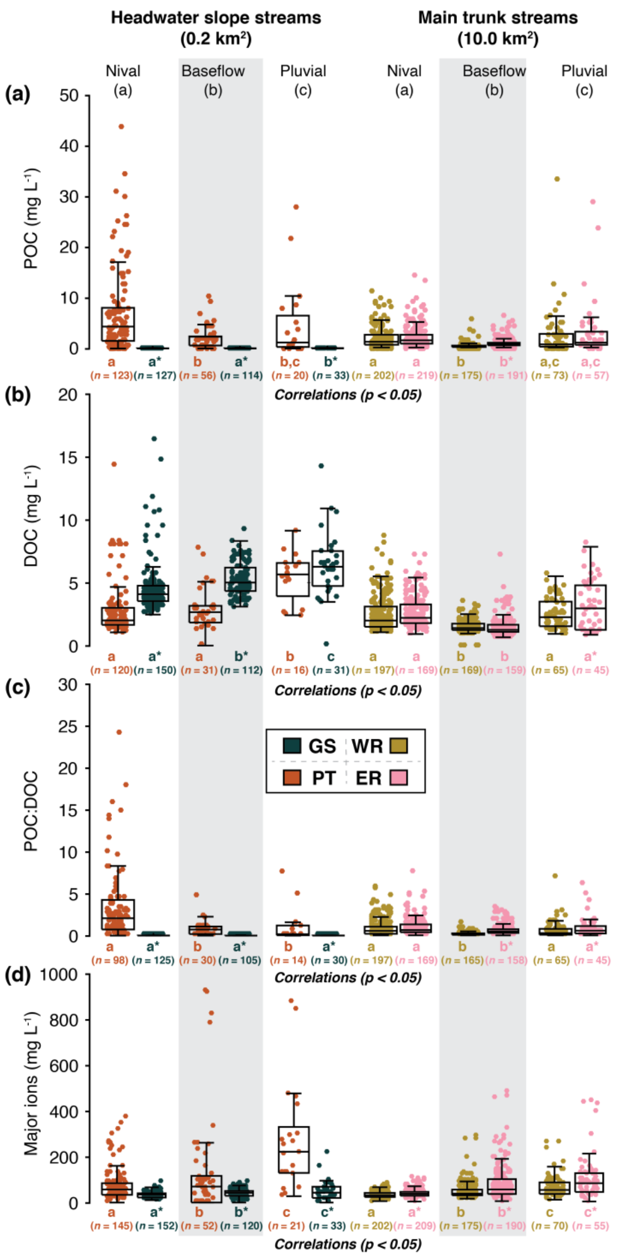

Supplementary Figure 10. Box plots showing carbon export in particulate (POC, a) and dissolved (DOC, b) form; ratios of POC to DOC export (c); and major ion concentrations (d). Each box plot shows the median (white line), interquartile range (colored boxes), upper and lower fences (black line "whiskers"; calculated as the upper/lower interquartile range limit ±1.5 times the interquartile range), and potential outlier observations (colored plus symbols). Statistically different differences between hydrological periods (two-sample t-test; $p < 0.05$) are noted using letters below each box plot (e.g. if the nival and baseflow period both have "a"s they are not significantly different, while a change of letter between periods, or unique letter combinations "a,b" vs. "c" are significantly different); asterisks signify significant ($p<0.05$) differences between the two watersheds for the same hydrological period. The number of observations used to calculate each box plot are noted beneath. All data are available in the Supplementary Data.

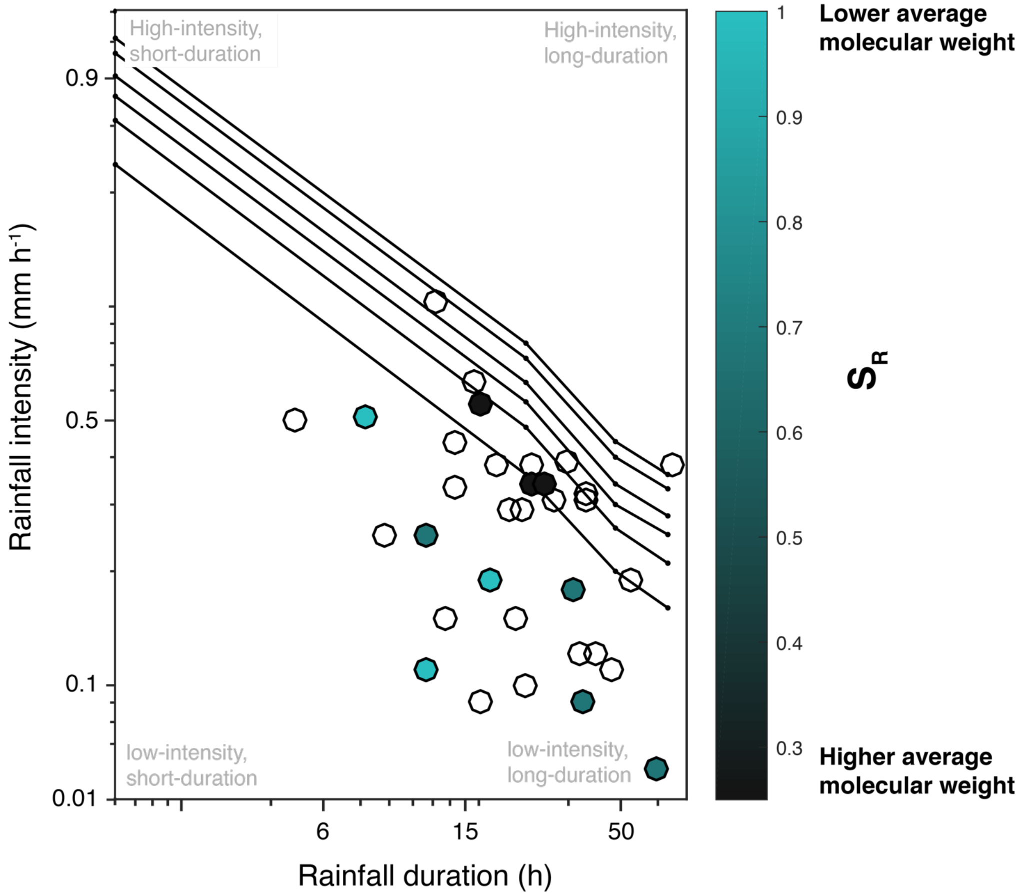

Supplementary Figure 11. The 72-hr rainfall intensity-duration-frequency curve overlain with a summary of spectral slope ratios (S_R) of chromophoric DOM, an indicator of average molecular weight (Helms et al., 2008) for pluvial data from all watersheds (GS, *n* = 6; PT, *n* = 5; WR, *n* = 10; ER, *n* = 4). Although limited, our data indicate that rainfall events with higher intensities, longer durations, and/or less frequent recurrence intervals lead to higher average molecular weight DOM in the stream networks during pluvial responses. All data are available in the Supplementary Data.

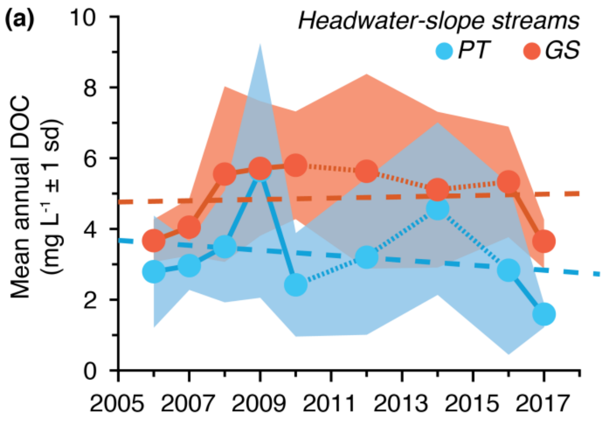

Supplementary Figure 12. Mean annual (2006-2017; ± 1sd shaded) DOC concentrations from headwater-slope streams: (a) Ptarmigan (PT; blue) and (b) Goose (GS; orange); and main watershed streams: (c) West river (WR; green) and (d) East river (ER; purple). Permafrost thaw-induced geomorphological disturbance resulted in a decline in interannual DOC concentrations post-disturbance at all watershed scales (PT, WR, ER; $p < 0.05$). In contrast, DOC concentrations from vegetated, physically undisturbed slopes increased during our study period (GS; $p < 0.05$).

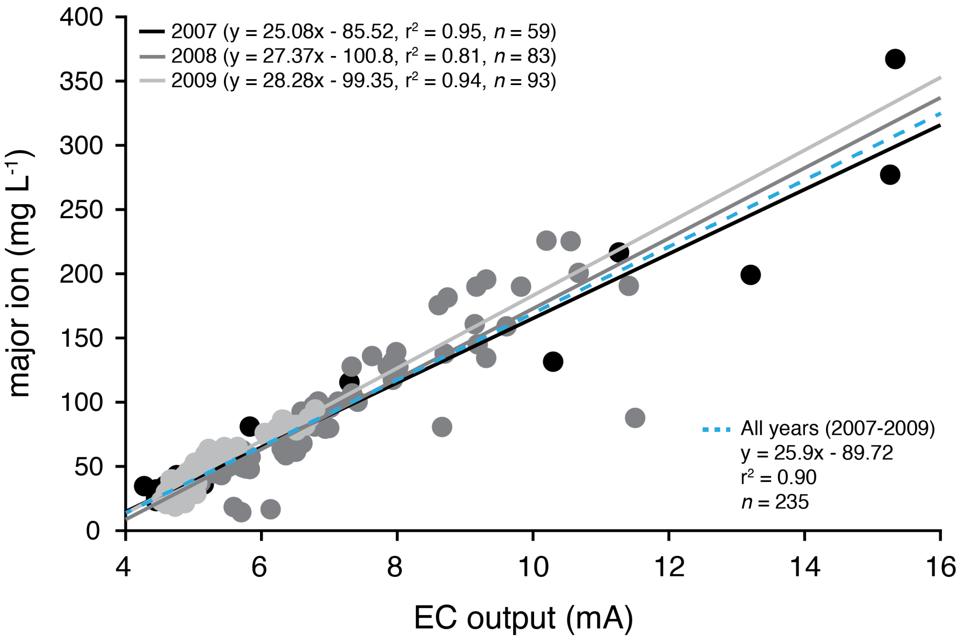

Supplementary Figure 13. Fitted linear correlations between in situ electrical conductivity (EC) output and measured concentrations of major ions for the East River, 2007-2009. Due to repeated failure of refrigerators used to store water samples prior to analysis (2012-2017), daily mean major ion concentrations were estimated from EC output, using the mean linear relationship for all years (blue line). One-way analysis of covariance indicates that the slopes are not significantly different from each other ($F(3,235) = 1.67$, $p > 0.1$).

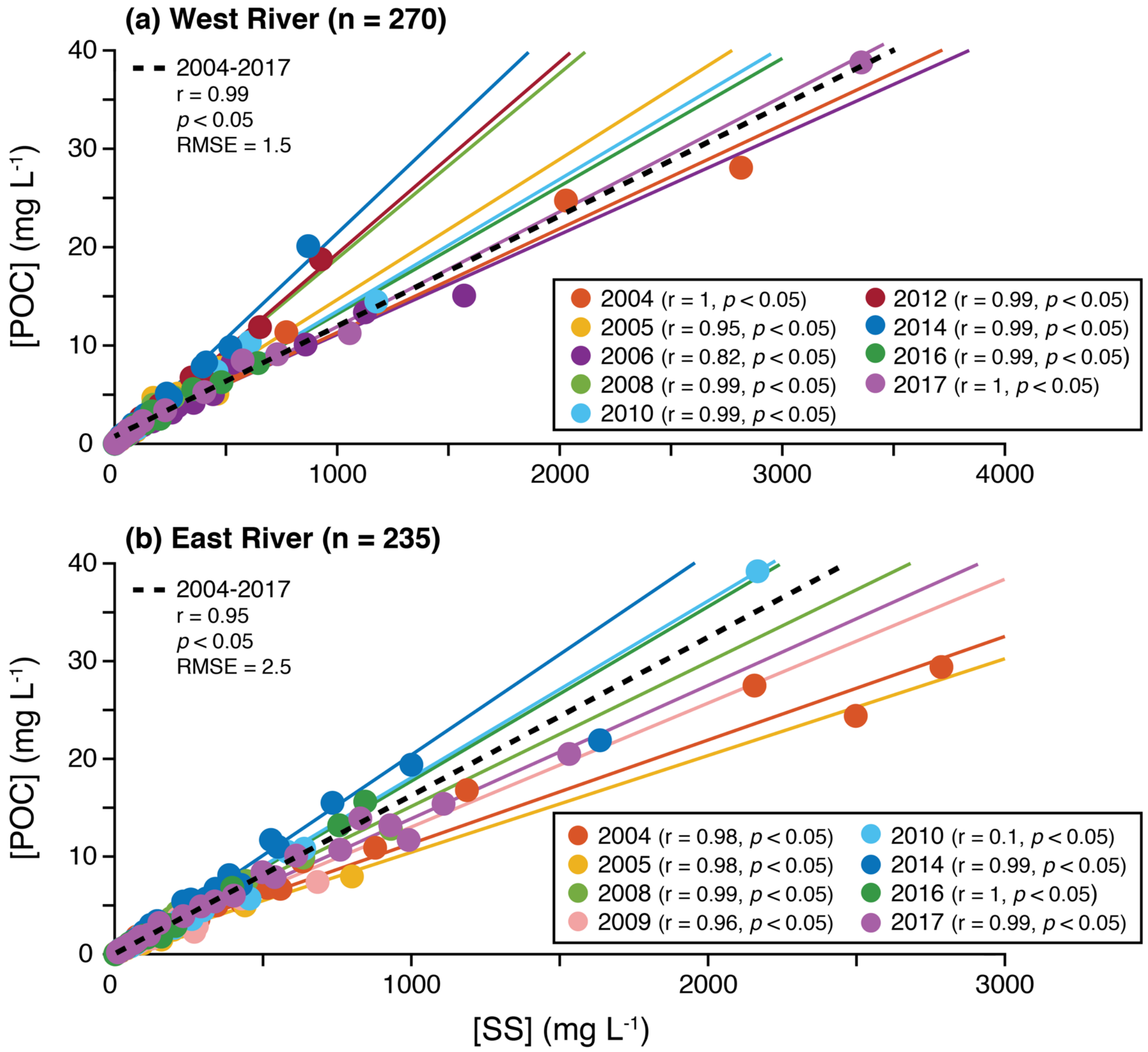

Supplementary Figure 14. Seasonal correlations between suspended sediment concentration ([SS]) and particulate organic carbon concentration ([POC]) for (a) WR: West river and (b) ER: East river, 2004-2017 (excluding 2011, 2013, 2015). Also shown is the correlation fit to all data (n = 235; 2004-2017; black dash line). Low root mean square errors (RMSE; standard deviation of residuals) show that the data is closely concentrated around the line of best fit but with more scatter at higher concentrations. Randomly selected replicate samples were run to estimate method uncertainty (Supplementary Figure 17; see Beel et al., 2020 for full details).

Supplementary Tables

Supplementary Table 2. Summary of hydrometeorological and biogeochemical instruments, dataloggers, and analytical methods used at CBAWO (2003-2019).

Parameter	Instrument	Instrument Accuracy	Datalogger	Years Used	Analytical method/material
Air Pressure	Omega-PRTEMP101	0.4%	Omega-PRTEMP101	2003-2006	
	Onset Hobo U20	0.3%	Onset Hobo U20	2007-2011	
	Onset CM50	0.4% (5 mb)	Onset CM50	2011-2019	
Air Temperature	Onset H8	0.2°C	Onset H8	2003-2006	Solar radiation shielded, 1.5 m above the surface
	Onset UA-003	0.1°C	Onset UA-003	2006-2019	
Rainfall	Davis Industrial	0.2 mm tip	Onset Hobo Event	2003-2006	1.5 m above the surface
			Onset UA-003	2006-2019	
Discharge (Q)	General Oceanics Flowmeter	1%	n/a	2003-2005	Velocity-area
	Swoffer 2100	1%	n/a	2006-2019	
Water Level (stage)	Sensym SCX	2 mm	Onset H8	2003	Stage-Q rating curve
	Omega CP-Level101	0.2% (0.5 mm)	Omega CR-PRTEMP101	2004-2005	
	Omega OM_CP Level 1000	0.2% (0.5 mm)	Omega OM-CP Level 1000	2006	
	Onset Hobo U20	0.3% (0.21 cm)	Onset Hobo U20	2007-2019	
Electrical Conductivity (EC)	Global Water WQ301	1%	Onset Hobo H22	2007-2019	EC-[TDS] rating curve
Suspended Sediment Concentration (SSC)	DH-48 Integrated water sampler	n/a	n/a	2003-2006	Volumetric filtration
	Teledyne ISCO 3700C Pump Sampler	n/a	n/a	2007-2019	
	n/a	n/a	n/a	2003	0.45-µm cellulose acetate filters
	n/a	n/a	n/a	2004-2019	1.0-µm glass fiber filters
Particulate Organic Carbon (POC)	LECO TruSpec CN Elemental Analyzer	0.0001 ppm	n/a	2004-2019	Dumas combustion reaction with non-dispersive infrared absorption
	n/a	n/a	n/a	2004-2019	Non-combusted, 1.0-µm glass fiber SSC filters
Major Ion Concentrations	Dionex ICS-3000	Most species: < 0.010 mg L^{-1}<br>Ca^{2+}: 0.053 mg L^{-1}<br>Mg^{2+}: 0.022 mg L^{-1}	n/a	2006-2019	Liquid ion chromatography (Anions, Cations). HCO_3^- determined by charge balance
	n/a	n/a	n/a	2006-2007	0.45-µm nitrocellulose membrane filters
	n/a	n/a	n/a	2008-2019	0.22-µm polyvinylidene fluoride filters

Supplementary Table 3. Pearson r correlation coefficients during nival periods. Correlations are considered statistically significant when $p < 0.05$.

	**Date**	**Stream power (W)**	**Q ($m^3\ s^{-1}$)**	**SSC (mg L^{-1})**	**POC (mg L^{-1})**	**DOC (mg L^{-1})**	**POC:DOC**	**TDN (mg L^{-1})**	**DOC:TDN**
**Date**	1.00		--	--	--	--	--	--	--
**Stream power (W)**	0.06	1.00	--	--	--	--	--	--	--
**Q ($m^3\ s^{-1}$)**	0.06	1.00*	1.00	--	--	--	--	--	--
**SSC (mg L^{-1})**	-0.05	-0.02	-0.02	1.00	--	--	--	--	--
**POC (mg L^{-1})**	-0.04	0.02	0.02	0.95*	1.00	--	--	--	--
**DOC (mg L^{-1})**	-0.16*	-0.09*	-0.09*	-0.18*	-0.19*	1.00	--	--	--
**POC:DOC**	0.00	0.02	0.02	0.92*	0.94*	-0.28*	1.00	--	--
**TDN (mg L^{-1})**	-0.19*	-0.06	-0.06	-0.12*	-0.12*	0.83*	-0.21*	1.00	--
**DOC:TDN**	-0.04	-0.07	-0.08	-0.17*	-0.19*	0.48*	-0.24*	0.02	1.00
**Major ions (mg L^{-1})**	0.08*	-0.14*	-0.14*	0.15*	0.12*	0.11*	0.08*	0.19*	-0.16*
**pH**	0.16*	-0.14*	-0.14*	0.02	0.03	-0.10*	0.06	-0.04	-0.13*
**GDD (°C)**	0.19*	-0.13*	-0.14*	0.02	0.03	-0.45*	0.13*	-0.32*	-0.34*
**River T (°C)**	-0.11*	-0.36*	-0.36*	-0.04	-0.08	-0.30*	-0.05	-0.26*	-0.12*
**Days since snowmelt initiation**	-0.06	-0.22*	-0.22*	-0.02	-0.02	-0.49*	-0.00	-0.42*	-0.26*
**α_{350} (m^{-1})**	-0.37*	-0.17	-0.16	0.26*	0.25*	0.80*	-0.17	0.75*	0.35*
**BIX**	-0.13	0.01	-0.00	0.01	-0.02	-0.36*	0.28*	-0.34*	-0.26*
**S_R**	0.14	-0.14	-0.14	-0.09	-0.07	-0.47*	0.33*	-0.47*	-0.20

*Statistically significant ($p < 0.05$) correlation

Supplementary Table 3 continued. Pearson r correlation coefficients during nival periods. Correlations are considered statistically significant when $p < 0.05$.

	Major ions (mg L^{-1})	pH	GDD (°C)	River T (°C)	Days since snowmelt initiation	α_{350} (m^{-1})	BIX	S_R
**Date**	--	--	--	--	--	--	--	--
**Stream power (W)**	--	--	--	--	--	--	--	--
**Q (m^3 s^{-1})**	--	--	--	--	--	--	--	--
**SSC (mg L^{-1})**	--	--	--	--	--	--	--	--
**POC (mg L^{-1})**	--	--	--	--	--	--	--	--
**DOC (mg L^{-1})**	--	--	--	--	--	--	--	--
**POC:DOC**	--	--	--	--	--	--	--	--
**TDN (mg L^{-1})**	--	--	--	--	--	--	--	--
**DOC:TDN**	--	--	--	--	--	--	--	--
**Major ions (mg L^{-1})**	1.00	--	--	--	--	--	--	--
**pH**	0.12*	1.00	--	--	--	--	--	--
**GDD (°C)**	-0.09*	0.20	1.00	--	--	--	--	--
**River T (°C)**	0.01	0.23*	0.71*	1.00	--	--	--	--
**Days since snowmelt initiation**	-0.04	0.18*	0.65*	0.74*	1.00	--	--	--
**α_{350} (m^{-1})**	0.43*	0.05	-0.31*	-0.40*	-0.39*	1.00	--	--
**BIX**	-0.01	-0.14	0.25*	0.34*	0.24*	-0.41*	1.00	--
**S_R**	-0.39*	0.11	0.12	0.37*	0.16	-0.50*	0.37*	1.00

*Statistically significant ($p < 0.05$) correlation

Supplementary Table 4. Pearson r correlation coefficients during baseflow periods. Correlations are considered statistically significant when $p < 0.05$.

	Date	Stream power (W)	Q ($m^3 s^{-1}$)	SSC (mg L^{-1})	POC (mg L^{-1})	DOC (mg L^{-1})	POC:DOC	TDN (mg L^{-1})	DOC:TDN
**Date**	1.00		--	--	--	--	--	--	--
**Stream power (W)**	-0.28*	1.00	--	--	--	--	--	--	--
**Q ($m^3 s^{-1}$)**	-0.28*	1.00*	1.00	--	--	--	--	--	--
**SSC (mg L^{-1})**	-0.22*	0.08	0.08	1.00	--	--	--	--	--
**POC (mg L^{-1})**	-0.20*	0.07	0.07	0.97*	1.00	--	--	--	--
**DOC (mg L^{-1})**	-0.02	-0.20*	-0.20*	-0.08	-0.12*	1.00	--	--	--
**POC:DOC**	-0.17*	0.07	0.08	0.85*	0.90*	-0.30*	1.00	--	--
**TDN (mg L^{-1})**	-0.22*	-0.13*	-0.13*	0.03	0.02	0.37*	-0.06	1.00	--
**DOC:TDN**	0.14*	-0.05	-0.05	-0.14*	-0.18*	0.70*	-0.36*	-0.23*	1.00
**Major ions (mg L^{-1})**	-0.01	-0.14*	-0.14*	0.03	0.06	0.01	0.03	0.39*	-0.27*
**pH**	0.15*	-0.28*	-0.27*	0.06	0.08	0.09	0.04	0.06	0.00
**GDD (°C)**	-0.26*	0.03	0.03	0.03	0.02	-0.10*	0.12*	0.12*	-0.24*
**River T (°C)**	-0.11*	-0.14*	-0.14*	-0.06	-0.06	0.24*	-0.04	0.15*	0.09
**Days since snowmelt initiation**	0.11*	-0.17*	-0.17*	-0.14*	-0.14*	-0.17*	-0.11*	0.13*	-0.34*
**α_{350} (m^{-1})**	-0.35*	-0.35*	-0.34*	-0.24*	-0.25*	0.89*	-0.32*	0.87*	0.61*
**BIX**	0.68*	0.16	0.16	-0.08	-0.08	-0.59*	0.04	-0.55*	-0.51*
**S_R**	0.24*	0.13	0.12	0.16	0.10	-0.40*	0.16	-0.37*	-0.37*

*Statistically significant ($p < 0.05$) correlation

Supplementary Table 4 continued. Pearson r correlation coefficients during baseflow periods. Correlations are considered statistically significant when $p < 0.05$.

	**Major ions (mg L^{-1})**	**pH**	**GDD (°C)**	**River T (°C)**	**Days since snowmelt initiation**	**α_{350} (m^{-1})**	**BIX**	**S_R**
**Date**	--	--	--	--	--	--	--	--
**Stream power (W)**	--	--	--	--	--	--	--	--
**Q (m^3 s^{-1})**	--	--	--	--	--	--	--	--
**SSC (mg L^{-1})**	--	--	--	--	--	--	--	--
**POC (mg L^{-1})**	--	--	--	--	--	--	--	--
**DOC (mg L^{-1})**	--	--	--	--	--	--	--	--
**POC:DOC**	--	--	--	--	--	--	--	--
**TDN (mg L^{-1})**	--	--	--	--	--	--	--	--
**DOC:TDN**	--	--	--	--	--	--	--	--
**Major ions (mg L^{-1})**	1.00	--	--	--	--	--	--	--
**pH**	0.34*	1.00	--	--	--	--	--	--
**GDD (°C)**	0.06	0.18*	1.00	--	--	--	--	--
**River T (°C)**	0.06	0.29*	0.84*	1.00	--	--	--	--
**Days since snowmelt initiation**	0.39*	0.09	-0.04	-0.10*	1.00	--	--	--
**α_{350} (m^{-1})**	0.06	0.39*	0.01	0.18	-0.17	1.00	--	--
**BIX**	0.01	-0.54*	-0.09	-0.37*	0.44*	-0.55*	1.00	--
**S_R**	-0.19	-0.11	0.18	-0.04	-0.25*	-0.33*	0.41*	1.00

*Statistically significant ($p < 0.05$) correlation

Supplementary Table 5. Pearson r correlation coefficients during pluvial (rainfall) periods. Correlations are considered statistically significant when $p < 0.05$.

	Date	Stream power (W)	Q ($m^3 s^{-1}$)	SSC (mg L^{-1})	POC (mg L^{-1})	DOC (mg L^{-1})	POC:DOC	TDN (mg L^{-1})	DOC:TDN
**Date**	1.00		--	--	--	--	--	--	--
**Stream power (W)**	-0.40*	1.00	--	--	--	--	--	--	--
**Q ($m^3 s^{-1}$)**	-0.40*	1.00*	1.00	--	--	--	--	--	--
**SSC (mg L^{-1})**	-0.27*	0.45*	0.45*	1.00	--	--	--	--	--
**POC (mg L^{-1})**	-0.29*	0.49*	0.50*	0.99*	1.00	--	--	--	--
**DOC (mg L^{-1})**	-0.11	-0.04	-0.03	0.07	0.06	1.00	--	--	--
**POC:DOC**	-0.31*	0.53*	0.54*	0.91	0.95*	-0.09	1.00	--	--
**TDN (mg L^{-1})**	-0.14	-0.11	-0.10	-0.04	-0.04	0.58*	-0.11	1.00	--
**DOC:TDN**	0.03	0.08	0.08	0.15	0.13	0.45*	-0.01	-0.32*	1.00
**Major ions (mg L^{-1})**	0.04	-0.13	-0.12	0.02	0.03	0.19*	-0.05	0.59*	-0.33*
**pH**	0.28*	-0.34*	-0.34*	-0.27*	-0.25*	-0.09	-0.22*	0.11	-0.24*
**GDD (°C)**	-0.02	-0.07	-0.07	0.04	0.03	-0.16	0.05	0.08	-0.15
**River T (°C)**	0.03	-0.28*	-0.29*	-0.09	-0.12	0.15	-0.18*	0.20*	0.02
**Days since snowmelt initiation**	0.23*	-0.17*	-0.17*	-0.04	-0.04	0.23*	-0.17*	0.27*	-0.06
**α_{350} (m^{-1})**	0.13	-0.17	-0.17	-0.18	-0.23	0.82*	-0.39*	0.78*	0.29
**BIX**	0.50*	-0.29	-0.29	-0.08	-0.10	-0.11	-0.07	-0.03	-0.19
**S_R**	-0.13	-0.16	-0.16	-0.20	-0.17	-0.36*	0.06	-0.25	-0.46*

*Statistically significant ($p < 0.05$) correlation

Supplementary Table 5 continued. Pearson r correlation coefficients during pluvial (rainfall) periods. Correlations are considered statistically significant when $p < 0.05$.

	**Major ions (mg L^{-1})**	**pH**	**GDD (°C)**	**River T (°C)**	**Days since snowmelt initiation**	**α_{350} (m^{-1})**	**BIX**	**S_R**
**Date**	--	--	--	--	--	--	--	--
**Stream power (W)**	--	--	--	--	--	--	--	--
**Q (m^3 s^{-1})**	--	--	--	--	--	--	--	--
**SSC (mg L^{-1})**	--	--	--	--	--	--	--	--
**POC (mg L^{-1})**	--	--	--	--	--	--	--	--
**DOC (mg L^{-1})**	--	--	--	--	--	--	--	--
**POC:DOC**	--	--	--	--	--	--	--	--
**TDN (mg L^{-1})**	--	--	--	--	--	--	--	--
**DOC:TDN**	--	--	--	--	--	--	--	--
**Major ions (mg L^{-1})**	1.00	--	--	--	--	--	--	--
**pH**	0.47*	1.00	--	--	--	--	--	--
**GDD (°C)**	0.12	0.36*	1.00	--	--	--	--	--
**River T (°C)**	0.22*	0.47*	0.77*	1.00	--	--	--	--
**Days since snowmelt initiation**	0.36*	0.32*	-0.05	0.18*	1.00	--	--	--
**α_{350} (m^{-1})**	0.63*	0.16	-0.31	-0.25	0.28	1.00	--	--
**BIX**	0.23	-0.15	-0.10	-0.37*	0.32	-0.05	1.00	--
**S_R**	-0.05	0.13	0.47*	0.40*	-0.33	-0.20	0.20	1.00

*Statistically significant ($p < 0.05$) correlation

Supplementary Table 6. Pearson r correlation coefficients versus antecedent weather conditions. Correlations are considered statistically significant when $p < 0.05$.

	Prior 48 hr		Prior week	
	**Mean T (°C)**	**Total P (mm)**	**Mean T (°C)**	**Total P (mm)**
**Date**	0.02	-0.02	0.05	-0.01
**Stream power (W)**	-0.18*	0.06*	-0.27*	-0.00
**Q (m^3 s^{-1})**	-0.19*	0.06*	-0.28*	-0.00
**SSC (mg L^{-1})**	-0.05	0.13*	-0.09*	0.11*
**POC (mg L^{-1})**	-0.05	0.12*	-0.10*	0.09*
**DOC (mg L^{-1})**	-0.32*	0.16*	-0.33*	0.13*
**POC:DOC**	0.03	-0.00	-0.05	-0.04
**TDN (mg L^{-1})**	-0.06	0.18*	-0.03	0.18*
**DOC:TDN**	-0.33*	0.04	-0.37*	-0.03
**Major ions (mg L^{-1})**	0.10*	0.18*	0.25*	0.25*
**pH**	0.27*	0.03	0.34*	-0.02
**GDD (°C)**	0.96*	-0.09*	0.78*	-0.08*
**River T (°C)**	0.77*	0.00	0.75*	0.07*
**Days since snowmelt initiation**	0.29*	0.32*	0.45*	0.49*
**α_{350} (m^{-1})**	-0.23*	0.15*	-0.30*	0.12
**BIX**	0.14*	0.10	0.27*	0.22*
**S_R**	0.20*	-0.01	0.29*	-0.10

*Statistically significant ($p < 0.05$) correlation

Supplementary Table 7. Analysis of variance (ANOVA) results for variables grouped by watershed (WS) and hydrological period (HP). Differences between groups are considered statistically significant when $p < 0.05$.

	**All data**		**By hydrological period (HP)**				**By watershed (WS)**		
	**By WS**	**By HP**	**PT**	**GS**	**WR**	**ER**	**Nival**	**Base flow**	**Pluvial**
**Date**	<0.01*	<0.01*	<0.01*	<0.01*	0.27	<0.01*	0.05	<0.01*	0.86
**Stream power (W)**	<0.01*	<0.01*	<0.01*	<0.01*	<0.01*	<0.01*	<0.01*	<0.01*	<0.01*
**Q (m^3 s^{-1})**	<0.01*	<0.01*	<0.01*	<0.01*	<0.01*	<0.01*	<0.01*	<0.01*	<0.01*
**SSC (mg L^{-1})**	<0.01*	<0.01*	<0.01*	<0.01*	<0.01*	<0.01*	<0.01*	<0.01*	<0.01*
**POC (mg L^{-1})**	<0.01*	<0.01*	<0.01*	<0.01*	<0.01*	<0.01*	<0.01*	<0.01*	<0.01*
**DOC (mg L^{-1})**	<0.01*	<0.01*	<0.01*	<0.01*	<0.01*	<0.01*	<0.01*	<0.01*	<0.01*
**POC:DOC**	<0.01*	<0.01*	0.10	0.10	<0.01*	0.01	<0.01*	<0.01*	<0.01*
**TDN (mg L^{-1})**	<0.01*	<0.01*	<0.01*	<0.01*	0.26	<0.01*	<0.01*	<0.01*	<0.01*
**DOC:TDN**	<0.01*	<0.01*	0.45	0.13	<0.01*	<0.01*	<0.01*	<0.01*	<0.01*
**Major ions (mg L^{-1})**	<0.01*	<0.01*	<0.01*	<0.01*	<0.01*	<0.01*	<0.01*	<0.01*	<0.01*
**pH**	<0.01*	<0.01*	<0.01*	0.01*	<0.01*	<0.01*	<0.01*	<0.01*	0.63
**GDD (°C)**	<0.01*	<0.01*	<0.01*	0.17	<0.01*	<0.01*	0.01*	<0.01*	0.55
**River T (°C)**	<0.01*	<0.01*	<0.01*	<0.01*	<0.01*	<0.01*	<0.01*	<0.01*	0.01*
**Days since snowmelt initiation**	<0.01*	<0.01*	<0.01*	<0.01*	<0.01*	<0.01*	<0.01*	<0.01*	0.65
**α_{350} (m^{-1})**	<0.01*	0.05	0.29	<0.01*	<0.01*	0.86	<0.01*	<0.01*	<0.01*
**BIX**	<0.01*	<0.01*	0.03*	0.34	<0.01*	<0.01*	<0.01*	<0.01*	0.46
**S_R**	<0.01*	0.035	0.90	0.92	0.14	0.41	0.02*	0.01*	0.19

*Statistically significant ($p < 0.05$) difference between groups

Supplementary Table 8. Two-sample Kolmogorov–Smirnov test results (p-values) comparing stream power distributions across sampled years in each watershed. Steam power distribution was considered significantly different between the two years at the $\alpha = 0.05$ level when $p < 0.05$.

	2006	***2007***	***2008***	***2009***	***2010***	***2012***	***2014***	***2016***	***2017***
**PT**									
2006	1.000	< 0.001*	< 0.001*	< 0.001*	< 0.001*	< 0.001*	< 0.001*	< 0.001*	< 0.001*
2007	< 0.001*	1.000	0.076	0.003*	0.249	0.001*	< 0.001*	< 0.001*	< 0.001*
2008	< 0.001*	0.076	1.000	0.087	0.371	< 0.001*	< 0.001*	< 0.001*	< 0.001*
2009	< 0.001*	0.003*	0.087	1.000	0.013*	< 0.001*	< 0.001*	< 0.001*	< 0.001*
2010	< 0.001*	0.249	0.371	0.013*	1.000	< 0.001*	< 0.001*	< 0.001*	< 0.001*
2012	< 0.001*	0.001*	< 0.001*	< 0.001*	< 0.001*	1.000	< 0.001*	< 0.001*	< 0.001*
2014	< 0.001*	< 0.001*	< 0.001*	< 0.001*	< 0.001*	< 0.001*	1.000	0.075	< 0.001*
2016	< 0.001*	< 0.001*	< 0.001*	< 0.001*	< 0.001*	< 0.001*	0.075	1.000	< 0.001*
2017	< 0.001*	< 0.001*	< 0.001*	< 0.001*	< 0.001*	< 0.001*	< 0.001*	< 0.001*	1.000
**GS**									
2006	1.000	< 0.001*	< 0.001*	< 0.001*	< 0.001*	< 0.001*	< 0.001*	< 0.001*	< 0.001*
2007	< 0.001*	1.000	< 0.001*	< 0.001*	< 0.001*	0.004*	< 0.001*	< 0.001*	0.004*
2008	< 0.001*	< 0.001*	1.000	0.017*	< 0.001*	< 0.001*	< 0.001*	< 0.001*	< 0.001*
2009	< 0.001*	< 0.001*	0.017*	1.000	0.047*	< 0.001*	< 0.001*	< 0.001*	< 0.001*
2010	< 0.001*	< 0.001*	0.002*	0.047*	1.000	< 0.001*	< 0.001*	< 0.001*	< 0.001*
2012	< 0.001*	0.004*	< 0.001*	< 0.001*	< 0.001*	1.000	< 0.001*	0.014*	0.110
2014	< 0.001*	< 0.001*	< 0.001*	< 0.001*	< 0.001*	< 0.001*	1.000	< 0.001*	< 0.001*
2016	< 0.001*	< 0.001*	< 0.001*	< 0.001*	< 0.001*	0.014*	< 0.001*	1.000	0.005*
2017	< 0.001*	0.004*	< 0.001*	< 0.001*	< 0.001*	0.110	< 0.001*	0.005*	1.000
**WR**									
2005	0.006*	< 0.001*	< 0.001*	< 0.001*	< 0.001*	< 0.001*	< 0.001*	< 0.001*	< 0.001*
2006	1.000	0.012*	< 0.001*	0.029*	< 0.001*	< 0.001*	0.001*	< 0.001*	< 0.001*
2007	0.012*	1.000	< 0.001*	0.534	< 0.001*	< 0.001*	0.059	< 0.001*	< 0.001*
2008	< 0.001*	< 0.001*	1.000	< 0.001*	0.196	0.326	0.005*	0.181	0.106
2009	0.029*	0.534	< 0.001*	1.000	< 0.001*	< 0.001*	0.130	< 0.001*	< 0.001*
2010	< 0.001*	< 0.001*	0.196	< 0.001*	1.000	0.271	0.145	0.303	0.093
2012	< 0.001*	< 0.001*	0.326	< 0.001*	0.271	1.000	0.123	0.470	0.049*
2014	0.001*	0.059	0.005*	0.130	0.145	0.123	1.000	0.005*	< 0.001*
2016	< 0.001*	< 0.001*	0.181	< 0.001*	0.303	0.470	0.005*	1.000	0.219
2017	< 0.001*	< 0.001*	0.106	< 0.001*	0.093	0.049*	< 0.001*	0.219	1.000
**ER**									
2005	0.002*	0.001*	< 0.001*	< 0.001*	< 0.001*	< 0.001*	< 0.001*	< 0.001*	< 0.001*
2006	1.000	0.086	0.007*	0.003*	0.043*	< 0.001*	0.007*	0.002*	0.177
2007	0.086	1.000	0.004*	0.015*	0.004*	< 0.001*	< 0.001*	0.005*	0.012*
2008	0.007*	0.004*	1.000	< 0.001*	< 0.001*	< 0.001*	0.011*	< 0.001*	0.001*
2009	0.003*	0.015*	< 0.001*	1.000	< 0.001*	< 0.001*	< 0.001*	< 0.001*	0.075
2010	0.043*	0.004*	< 0.001*	< 0.001*	1.000	0.241	0.070	0.950	0.001*
2012	< 0.001*	< 0.001*	< 0.001*	< 0.001*	0.241	1.000	0.032*	0.185	< 0.001*
2014	0.007*	< 0.001*	0.011*	< 0.001*	0.070	0.032*	1.000	0.104	< 0.001*
2016	0.002*	0.005*	< 0.001*	< 0.001*	0.950	0.185	0.104	1.000	< 0.001*
2017	0.177	0.012*	0.001*	0.075	0.001*	< 0.001*	< 0.001*	< 0.001*	1.000

* Statistically significant differences in stream power distributions between the two years
