# Supplementary material for: Emerging dominance of summer rainfall driving High Arctic terrestrial-aquatic connectivity
Source: Nat Commun. 2021 Mar 4;12:1448. doi: 10.1038/s41467-021-21759-3 (PMC7933336; doi:10.1038/s41467-021-21759-3)
Supplement: Supplementary file 9 — Description of Additional Supplementary Files [file 41467_2021_21759_MOESM9_ESM.docx]

Description of additional supplementary files

Title: Supplementary Data 1

Description: Mean daily discharge, river temperature, stream power and concentrations of total suspended sediments, dissolved and particulate organic carbon, total dissolved nitrogen, major ions, and dissolved inorganic nitrogen (2003-2017)

Title: Supplementary Data 2

Description: Daily fluvial fluxes (2003-2017)

Title: Supplementary Data 3

Description: Rainfall events and pluvial runoff and material fluxes (2003-2017)

Title: Supplementary Data 4

Description: Daily air temperature and rainfall (2003-2019)

Title: Supplementary Data 5

Description: Optical dissolved organic matter indices (2012-2017)

Title: Supplementary Data 6

Description: Daily growing degree day (GDD; 2003-2019)
